# Relationship Between Schizotypal Traits, Emotion Regulation, and Negative Affect in Children: A Network Analysis

**DOI:** 10.1093/schbul/sbae172

**Published:** 2025-03-04

**Authors:** Qian Ren, Tian-xiao Yang, Yi Wang, Simon S Y Lui, Raymond C K Chan

**Affiliations:** Neuropsychology and Applied Cognitive Neuroscience, CAS Key Laboratory of Mental Health, Institute of Psychology, Chinese Academy of Sciences, Beijing 100101, China; Department of Psychology, University of Chinese Academy of Sciences, Beijing 100101, China; Neuropsychology and Applied Cognitive Neuroscience, CAS Key Laboratory of Mental Health, Institute of Psychology, Chinese Academy of Sciences, Beijing 100101, China; Department of Psychology, University of Chinese Academy of Sciences, Beijing 100101, China; Neuropsychology and Applied Cognitive Neuroscience, CAS Key Laboratory of Mental Health, Institute of Psychology, Chinese Academy of Sciences, Beijing 100101, China; Department of Psychology, University of Chinese Academy of Sciences, Beijing 100101, China; Department of Psychiatry, School of Clinical Medicine, The University of Hong Kong, Hong Kong Special Administrative Region 999077, China; Neuropsychology and Applied Cognitive Neuroscience, CAS Key Laboratory of Mental Health, Institute of Psychology, Chinese Academy of Sciences, Beijing 100101, China; Department of Psychology, University of Chinese Academy of Sciences, Beijing 100101, China

**Keywords:** schizotypal traits, negative affect, emotion regulation, network analysis

## Abstract

**Background and Hypothesis:**

Evidence suggests that emotion regulation is related to schizotypal traits and negative affect in adults. Few studies examined the interplay among these constructs in school-aged children. We examined the complex relationship between schizotypal traits, emotion regulation, and negative affect in children aged 9–12 years.

**Study Design:**

One-thousand-and-nineteen children completed the Schizotypal Personality Questionnaire—children (SPQ-C), the Depression Anxiety Stress Scales (DASS-21), and the Emotion Regulation Questionnaire for Children and Adolescence (ERQ-CA). Using subscales of these measures as nodes, we estimated a partial correlation network. We estimated a Directed Acyclic Graph to explore the putative directional relationship between schizotypal traits, emotion regulation, and negative affect. Node and bridge centrality indices were estimated.

**Results:**

We found positive correlations between schizotypal dimensions and negative affect (depressed mood, anxiety, and stress) in the network. Emotion suppression was positively correlated with interpersonal and disorganized schizotypal dimensions, and negative affect. Emotion reappraisal was positively correlated with the cognitive–perceptual dimension and negatively correlated with interpersonal schizotypal traits, depressed mood, and stress. Stress showed higher strength than all nodes except depressed mood, and stress showed the highest expected influence (EI). The Bayesian network revealed that schizotypal traits appeared to be driven by stress. Network comparisons preliminarily showed higher EI for emotion reappraisal in girls’ than boys’ networks, and significant impacts of age and schizotypy levels on network patterns.

**Conclusion:**

Children with higher levels of schizotypal traits may have more negative affect and suppression. Stress appears to drive schizotypal traits.

## Introduction

Schizotypy refers to the latent psychological organization reflecting the liability to schizophrenia,^1–3^ and encompasses the cognitive–perceptual (positive schizotypy), interpersonal (negative schizotypy), and disorganized (disorganized schizotypy) dimensions. Schizotypal traits are continuous phenotypes, spanning from subclinical to clinical entities of schizophrenia spectrum disorders (SSD).^4^ Schizotypal traits can manifest in typical-developing children at the age of 5–6,^5–7^ and predict future risk for psychosis in adulthood.^8^ Studying childhood schizotypal traits can advance our understandings as to how SSD emerge.^7,9^

Negative affect (ie, depressed mood, anxiety, and stress) is prevalent in people with schizotypal traits,^10,11^ and aggravates the risk for psychosis transition.^12–16^ At the dimension level, while some findings showed that positive rather than negative schizotypy showed a stronger association with depressed mood and anxiety,^17^ others showed that disorganized schizotypy instead of positive schizotypy showed a strong association with NA (negative affect).^18^ Notably, the research found that the correlation between positive schizotypy and NA disappeared after controlling for disorganized schizotypy.^19,20^ These prior findings urged further research on schizotypy as separate dimensions. The directionality between schizotypal traits and NA remains unclear, as prior research supported both directions.^21^ SSD individuals might exhibit NA in response to non-stressful situations,^21,22^ while NA could also worsen psychotic experiences.^4,23^

Emotion regulation refers to the processes to modify the frequency, intensity, and duration of emotional experiences,^24^ and has 2 broad types—suppression and reappraisal.^25^ The former type reduces the expressions of explicit behaviors induced by emotions, while the latter type reinterprets the situations that have elicited the experienced emotions.^25^ In typical-developing children, frequent emotion suppression is related to higher NA,^26^ while frequent reappraisal is related to lower NA.^27^ SSD individuals typically have difficulties in emotion regulation.^28,29^ In adult populations, the 3 schizotypal dimensions were correlated positively with suppression.^30^ People prone to develop delusions (ie, positive schizotypy) were impaired in reappraisal rather than suppression.^31^ However, reappraisal might reduce NA in people with early psychosis.^32,33^

Schizotypal traits, emotion regulation, and NA are intricately interrelated. It is necessary to take into account emotion regulation, when we clarify the relationship between schizotypal traits and NA. Traditional statistical models view symptoms as manifestations of the latent disease, which bear a priori assumption of causality. Network analysis, however, views mental disorders as complex systems of interacting symptoms, employing a data-driven approach without any a priori hypothesis.^34,35^ The key advantage of network analysis allows us to examine the independent relationships between 2 variables while controlling for others.^36,37^ Moreover, network analysis provides centrality indices to illustrate the interconnectedness of variables^35,38^ and bridge centrality indices to identify variables that connect different theoretical communities.^39^ While partial correlation networks are “undirected,” Bayesian networks can employ Directed Acyclic Graphs (DAGs) to represent the overall dependence structure, for exploring possible causal directions.^40–43^ Bayesian networks are useful to study psychosis.^40–43^ For example, Moffa et al^44^ employed DAGs and found that sexual abuse and bullying apparently drove affective symptoms via worry, leading to hallucinations. Although not “confirming” causality, DAGs could unveil complex mechanisms of psychotic disorders, and guide further research on causality issues.^44,45^

This study aimed to unveil the complex interrelationship between schizotypal traits, NA, and emotion regulation in children, at the dimension level. We also tentatively explored the directional dependency between schizotypal traits and NA using Bayesian networks. We preliminarily examined the effects of age, gender, and high/low schizotypal traits using network comparison tests (NCTs). Based on previous research, we hypothesized that NA would correlate positively with the cognitive–perceptual and disorganized dimensions of schizotypal traits, rather than with the interpersonal dimension. Second, schizotypal trait dimensions would correlate negatively with emotion reappraisal, but positively with emotion suppression. Third, NA would correlate negatively with reappraisal, but positively with suppression. Given the previous mixed findings, we did not make any specific hypothesis on network comparisons, nor assume specific directional dependency between variables.

## Method

### Participants

We recruited 1333 primarily school students in grades 4–6 from 2 schools in Jiangxi and Shandong provinces. Data were collected via a standardized procedure, so that participants completed self-report questionnaires in quiet environments, where trained assistants were ready to address participants’ questions. Data gathered from 314 children were excluded due to missing data (*n* = 295), encoding errors (*n* = 16) and aged >13 (*n* = 2), aged < 9 (*n* = 1). Our final sample comprised 1019 participants with complete and valid data (boys = 540, girls = 479, *M*_age_ = 10.39, SD_age_ = 0.96). Table 1 shows participants’ demographics. This study was approved by the Ethics Committee of the Institution of Psychology, Chinese Academy of Sciences (protocol number: H20033). Participants’ guardians provided written informed consent.

**Table 1.** Descriptive Characteristics of the Entire Sample

Variables and the dimensions (*N* = 1019)	Minimum	Maximum	Mean	SD	Skewness	Kurtosis
Age (y)	9	12	10.39	0.96	0.06	−0.95
Length of education (y)	4	6	5	0.82	−0.01	−1.52
SPQ-C total score	0	22	6.28	4.62	0.51	−0.41
SPQ-C cognitive perceptual	0	8	2.83	2.08	0.37	−0.73
SPQ-C interpersonal	0	8	2.39	2.06	0.57	−0.55
SPQ-C disorgani zed	0	6	1.06	1.37	1.40	1.38
ERQ-CA total score	10	50	28.62	9.57	−0.15	−0.53
ERQ-CA reappraisal	6	30	18.33	6.72	−0.16	−0.89
ERQ-CA suppression	4	20	10.28	4.32	0.30	−0.73
DASS-21 total score	0	63	10.96	10.95	1.49	2.47
DASS-21 depression	0	21	2.93	3.89	1.90	3.80
DASS-21 anxiety	0	21	4.11	4.08	1.30	1.52
DASS-21 stress	0	21	3.91	4.05	1.38	1.97

Abbreviations: DASS-21, The Depression Anxiety Stress Scales; ERQ-CA, Emotion Regulation Questionnaire for Children and Adolescent; SPQ-C, Schizotypal Personality Questionnaire—Children.

### Measurements

#### Schizotypal Traits

The self-report Schizotypal Personality Questionnaire—children (SPQ-C)^46,47^ has 22 items and 3 (cognitive–perceptual, interpersonal, and disorganized) subscales. Each item is rated on a yes/no choice. In this study, the Cronbach’s alpha coefficients for the total score, the cognitive–perceptual, the interpersonal, and the disorganized subscale scores were 0.84, 0.69, 0.71, and 0.67, respectively. The SPQ-C has been validated in the Chinese setting,^46,47^ applicable to children aged 8–16 years.^46,47^

#### Emotion Regulation

We administered the modified version of the self-report Emotion Regulation Questionnaire for Children and Adolescence (ERQ-CA),^48^ which has demonstrated good reliability and validity in Chinese primary school students aged 9–12.^49^ Each item is rated on a 5-point Likert scale. The Cronbach’s alpha coefficients for the ERQ-CA suppression and reappraisal were 0.67 and 0.80, respectively, in this study.

#### Depression, Anxiety, and Stress

The short-form version of the self-report Depression Anxiety Stress Scales (DASS-21) was used to measure negative affect.^50^ DASS-21 comprises 3 factors, including anxiety (7 items), depression (7 items), and stress (7 items). Each item is rated on a 4-point Likert scale. DASS-21 has been validated in Chinese children and adolescents with good reliability and validity.^51^ The Cronbach’s alpha coefficients for the total score, and depression, anxiety, and stress subscale scores were 0.92, 0.82, 0.77, and 0.79, respectively, in this study.^51^

### Data Analysis

#### Descriptive Analysis

Descriptive and comparative analyses were conducted using the SPSS 22 software. Independent sample *t*-test (2-tailed) compared the gender-stratified groups in age, emotion regulation, schizotypal traits, and negative affect. The threshold for significance was set at *P* < .05. Effect size was estimated as Cohen’s *d*. We examined the kurtosis and skewness of all measurements. In this study, we did not remove participants with extreme values of these scales as “outliers,” because they might represent people with high levels of schizotypy who had highly negative affect and highly altered emotion regulation. However, as sensitivity analysis, we repeated the same analysis after the removal of outliers,^52^ as shown in Supplementary Material 2.

#### The Network Analysis for the Entire Sample

We constructed network analysis according to Epskamp, Borsboom, and Fried’s guidelines^35,53^ using the qgraph package in R version 4.2.3 (R script is shown in Supplementary Material 3). First, variables were transformed into *z*-scores for comparability and standardization. For the entire sample, we used the SPQ-C subscale scores, ERQ-CA subscale scores, and DASS-21 subscale scores as nodes in the network. The edges represented the partial correlations between each pair of nodes, after controlling for all other nodes. Given the variables (nodes) followed a non-normal distribution, Spearman’s partial correlations were used to estimate the network. The Least Absolute Shrinkage and Selection Operator combined with Extended Bayesian Information Criteria model selection was applied, to shrink relatively weak correlations to zero, and limit “false-positive” edges. To balance the inclusion of genuine and spurious edges, we set the tuning parameter to its default value of 0.5.^35^ Edges in blue indicated positive correlations; edges in red indicated negative correlations. Thicker edges indicated stronger correlations between nodes. Node centrality indices (strength, betweenness, and closeness), predictability, and expected influence (EI) for each node were estimated. We further estimated the stability of the centrality indices, and the accuracy of edge weights, using the R bootnet package.

#### Bridge Centrality Indices

We estimated bridge centrality to identify the bridging nodes that connect different communities in the network, using the networktools package in R.^39^ Bridge strength, bridge closeness, bridge betweenness, and bridge EI (1-step) were estimated. These indices quantify the importance of a node in linking other communities. For instance, bridge strength refers to the sum of connectivity between a node and all nodes that are not in the same community within the network. To avoid confirmation biases, we employed an 80th percentile cutoff to select bridge nodes.^39^

#### Network Comparison

We employed the NCT in R,^54^ and assessed the impact of gender, age group, and schizotypy level (high/low) on network patterns. For gender differences, we constructed networks for gender-stratified groups, and compared them in terms of network structure, global strength, edge weights, and centrality indices using 2-tailed permutation tests with 10 000 iterations. The invariance of network structure was tested by evaluating the maximum difference between connection strength matrices. The global strength of the networks was tested by the sum of all absolute connection strengths between all node pairs. Given its broad age range, we divided our sample into 2 groups, namely a subgroup aged 9–10 (*n* = 544) and another subgroup aged 11–12 (*n* = 475). Using NCT, we preliminarily examined the potential effect of age on the interrelationship between variables of interest. We further categorized our sample into the high (*n* = 452) and low (*n* = 483) schizotypal groups, using a median-split of the SPQ-C total score,^55^ and conducted NCT.

#### Bayesian Network

We tentatively explored the directions of the relationship between schizotypal traits and negative affect, using the bnlearn package in R for DAG network analysis.^40,56,57^ This method has already been used to explore causal relationships related to schizophrenia.^40–42^ In DAGs, the lack of a direct edge indicates that 2 nodes are independent, or conditionally independent after accounting for the other nodes, whereas the presence of a direct edge indicates a dependence between 2 nodes after conditioning on any other nodes.^55,58^

Following the guidelines,^59^ we employed the Hill-Climbing (HC) algorithm,^59^ a score-based greedy search method, to obtain the Bayesian network structure that best fits the data, based on the Bayesian Information Criterion. We applied the HC algorithm to 1000 bootstrap samples for stability, with each DAG inference including 5 random restarts and 10 perturbations per restart to reduce the risk of local maxima^60^ (for details, see McNally et al^60^). The edges in the DAG were determined by arc strength, reflecting their frequency in the bootstrap networks.^59^ We retained edges with a frequency of at least 85% in the final “consensus” network. Before this, all learned DAGs were transformed into Completed Partially Directed Acyclic Graphs.^56,61^ After determining the edges, we assessed the frequency of each edge direction in the bootstrap networks. If an edge direction was estimated over 50% of the networks, it was included in the final network.^59,60^

It should be borne in mind that the influence of unmeasured confounders, such as common causes of 2 or more nodes in the DAG, could potentially undermine the DAG results.^55,58^ However, it is practically impossible for DAG to include all potential variables and confounders,^45,62^ therefore the DAG results must be interpreted with caution.

## Results

### Regularized Partial Correlation Network

**Figure 1.**
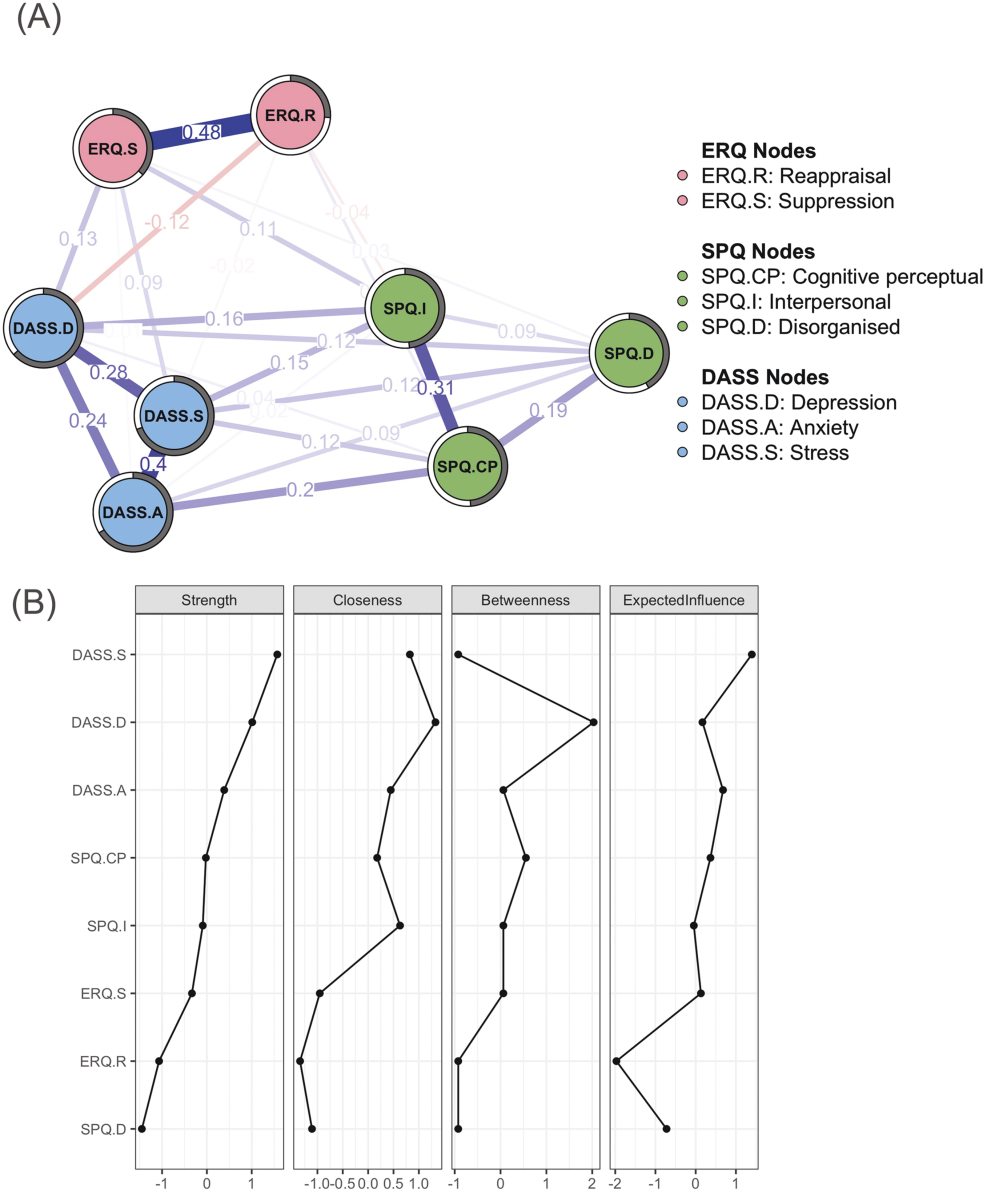
(A) Regularized Partial Correlation Network for the Whole Sample. Each Node Represents a Variable. Each Edge Represents the Partial Correlation Between Two Nodes Controlled for All Other Nodes. Thicker Lines Represent Stronger Connections. The Value of Each Edge Represents the Strength of the Partial Correlations. Positive Edge Values Indicate Positive Partial Correlations and Negative Edge Values Indicate Negative Partial Correlations. The Pie Chart Around Each Node Represents the Predictability Values, Which Indicate Prediction of a Specific Node by Other Nodes in the Network. (B) The Standardized Centrality Estimates of the Whole-Sample Network. Abbreviations: DASS.A, Anxiety Dimension of Depression Anxiety Stress Scales; DASS.D, Depression Dimension of Depression Anxiety Stress Scales; DASS.S, Stress Dimension of Depression Anxiety Stress Scales; ERQ.R, Reappraisal Dimension of Emotion Regulation Questionnaire; ERQ.S, Suppression Dimension of Emotion Regulation Questionnaire; SPQ.CP, Cognitive Perceptual Dimension of Schizotypal Personality Questionnaire; SPQ.D, Disorganized Dimension of Schizotypal Personality Questionnaire; SPQ.I, Interpersonal Dimension of Schizotypal Personality Questionnaire

The regularized partial correlation network is shown in Figure 1A. After controlling for the inter-relationships between nodes, we observed positive partial correlations between the cognitive–perceptual (SPQ-CP), interpersonal (SPQ-I), and disorganized (SPQ-D) dimensions of schizotypal traits, and negative affect (ie, depressed mood, anxiety, and stress) (*edge values* = 0.02–0.20). The difference test for edge weights (see Supplementary Figure S1.2) showed that the correlation between SPQ-CP and anxiety was significantly greater than that between SPQ-D, SPQ-I, and anxiety.

Moreover, the SPQ-I and SPQ-D nodes were positively correlated with emotion suppression (*edge values* = 0.11 and 0.03, respectively). The SPQ-CP node was positively correlated with emotion reappraisal (*edge value* = 0.05), and the SPQ-I node was negatively correlated with emotion reappraisal (*edge value* = −0.04). Partial positive correlations were found between emotion suppression and nodes of negative affect (ie, depressed mood, anxiety, and stress) (*edge values* = 0.01–0.13). Partial negative correlations were found between emotion reappraisal, depressed mood, and stress (*edge values* = −0.12 and −0.02, respectively).

The relationships between schizotypal traits and the negative affect observed remained stable in the sensitivity analysis. Additionally, there were no significant differences in NCTs between the sensitivity analysis network (excluding “outliers”) and the present network (see Supplementary Material 2 for details), indicating the stability of the results.

### Network Inference

Centrality indices are shown in Figure 1B. Based on the difference test for node centrality in Supplementary Figure S1.3, stress had significantly higher strength than all other nodes (except depressed mood). Stress had significantly higher EI than other nodes. Depressed mood displayed higher closeness centrality than emotion suppression, emotion reappraisal, and SPQ-D, but was not significantly higher than the remaining nodes. The predictability of nodes ranged from 26% (emotion reappraisal) to 69% (stress), with an average of 50%. Supplementary Table S1.1 details the node centrality indices. Sensitivity analyses showed similar results, indicating the stability of the results.

### Bridging Centrality Indices

Bridge centrality indices are displayed in Supplementary Figure S1.4. Using an 80th quantile cutoff, depressed mood and stress had the highest bridge strength, stress, and SPQ-I had the highest bridge EI (1-level), and depressed mood and SPQ-I showed the highest bridge closeness. However, according to the difference test for bridge node centrality (see Supplementary Figure S1.5), most of the differences between nodes failed to reach statistical significance, except that the bridge EI of emotion reappraisal was significantly lower than all other nodes. Sensitivity analyses showed similar results, indicating the stability of the results.

### NCT Results Between Boys and Girls

Independent sample *t*-tests (see Supplementary Table S1.4) showed that girls scored higher than boys in all dimensions of schizotypal traits, emotion suppression and reappraisal, and NA (depressed mood, anxiety, and stress). Supplementary Figure S1.7 shows the regularized partial correlation networks for boys and girls. NCT found the 2 gender-stratified networks had comparable network structure (ie, the maximum difference in edge weights was 0.15, *P* = .22) and global strength (ie, the strength difference was 0.29, *P* = .17, global strength = 3.44 for boys, and 3.73 for girls). Regarding the centrality indices, emotion reappraisal demonstrated a larger EI (*P* < .05) in the girls’ rather than boy’s network. NCT also found comparable edge weights between the 2 gender-stratified networks (all *P*s > .05). However, in the sensitivity analysis excluding the potential outliers, the independent sample *t*-tests showed that the gender differences in the SPQ-CP and SPQ-D dimensions were not significant. Moreover, the gender difference in emotion reappraisal in terms of EI disappeared.

### NCT Results Between the Two Age Groups

For age effect, independent sample *t*-tests (see Supplementary Table S1.5) showed that children aged 11–12 scored higher than children aged 9–10 in SPQ-D and depressed mood. The regularized partial correlation networks for subgroups aged 9–10 and 11–12 are shown in Supplementary Figure S1.8. NCT showed significant differences in global strength (ie, the strength difference was 0.41, *P* < .05, with a global strength of 3.24 for the subgroup aged 9–10 and 3.65 for the subgroup aged 11–12), and the network structure (ie, the maximum difference in edge weights was 0.21, *P* < .01). Additionally, NCT found children aged 11–12 having a stronger correlation between SPQ-CP and anxiety, compared with children aged 9–10 (*P* < .05). After sensitivity analysis, the findings regarding the subgroup difference in anxiety—SPQ-CP (*P* < .05) correlation, and network structure (*P* < .01) remained the same.

### NCT Results Between the High and Low Schizotypy Subgroups

As shown in Supplementary Table S1.6, the high schizotypy group exhibited higher levels of schizotypal traits, NA, and used both higher emotion suppression and reappraisal than the low schizotypy group. Supplementary Figure S1.9 displays the regularized partial correlation networks for 2 groups. NCT showed significant differences in the network structure (ie, the maximum difference in edge weights was 0.34, *P* < .001), but not global strength (ie, the strength difference was 0.57, *P* = .12, with a global strength of 2.70 for the high schizotypy subgroup and 3.28 for the low schizotypy subgroup).

Moreover, the low schizotypy subgroup demonstrated higher strength in SPQ-CP, higher strength in emotion suppression, and higher strength in reappraisal, as well as higher EI of SPQ-CP and higher EI of emotion reappraisal than the high schizotypy subgroup (*P*s < .05). NCT also found significant differences in edge weights after FDR (False Discovery Rate) correction, including SPQ-CP—Reappraisal (*P* < .05), SPQ-CP—Suppression (*P* < .05), SPQ-D—DASS Depressed mood (*P* < .05), Reappraisal—Suppression (*P* < .01), SPQ-CP—SPQ-I (*P* < .05), and SPQ-I—SPQ-D (*P* < .05). Sensitivity analyses showed stable results, implicating that the between-subgroup differences in the network structure, EI of emotion reappraisal, and the Reappraisal—Suppression correlation remained significant (*P*s < .05).

### Network Stability and Accuracy

**Figure 2.**
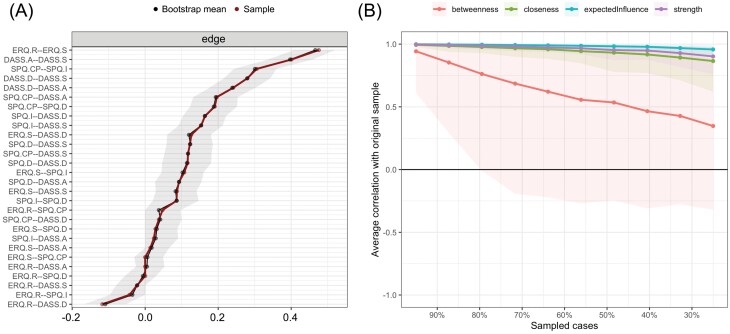
(A) Bootstrapped 95% CIs of Edge Weights of the Whole-Sample Network. The Shaded Area Represents the Bootstrapped CI and the Sample Line Represents the Edge Weight. (B) The Stability of Centrality Indicators Computed by Case-Dropping Bootstrap. The Lines Represent the Average Correlation Between the Central Indicators Estimated From the Bootstrapping Subsamples and the Central Indicators Estimated From the Original Sample. The Areas Represent a Range From 2.5th to 97.5th Quantiles. Abbreviations: DASS.A, Anxiety Dimension of Depression Anxiety Stress Scales; DASS.D, Depression Dimension of Depression Anxiety Stress Scales; DASS.S, Stress Dimension of Depression Anxiety Stress Scales; ERQ.R, Reappraisal Dimension of Emotion Regulation Questionnaire; ERQ.S, Suppression Dimension of Emotion Regulation Questionnaire; SPQ.CP, Cognitive Perceptual Dimension of Schizotypal Personality Questionnaire; SPQ.D, Disorganized Dimension of Schizotypal Personality Questionnaire; SPQ.I, Interpersonal Dimension of Schizotypal Personality Questionnaire

Figure 2A shows relatively narrow bootstrapped CIs, suggesting that edge weights were reliable.^37^ The recommended threshold of the correlation stability (CS) coefficients is 0.5.^37^ The CS coefficients for strength, closeness, and EI were 0.75, 0.67, and 0.75, respectively, suggesting stability. The CS coefficients for bridge strength, bridge closeness, and bridge EI were 0.60, 0.52, and 0.75, respectively, suggesting stability (see Supplementary Figure S1.6). However, the CS coefficients for betweenness and bridge betweenness were both below 0.05, indicating low stability.

### Bayesian Network

Bayesian network could explore the directional relationship between schizotypal traits and negative affect. Nodes located in the upstream (near the top) of the DAG network were sources of activation to drive other nodes. Supplementary Figure S1.10 shows the DAG for the whole sample. Stress was an influential node to drive the 3 dimensions of schizotypal traits. The depressed mood had an edge pointing toward SPQ-I and SPQ-D. The SPQ-CP node had an edge pointing toward anxiety.

The SPQ-I had an edge pointing toward emotion suppression. In the sensitivity analysis, some findings remained consistent. Stress remained a significant node driving all 3 dimensions of schizotypal traits, depressed mood had an edge pointing to SPQ-D, and SPQ-I had an edge pointing toward emotion suppression. We used an 85% threshold to select edges, and a 50% threshold to determine direction. However, different thresholds were used in previous studies to select edges (eg, 50%,^63^ 75%,^40^ and 85%^57,60,64^), which could affect the results. Thus, we provided details for any edge with an arc strength greater than 50% for reference in Supplementary Tables S1.7–S1.11.

Given the significant differences found in the network comparisons between age groups and high/low schizotypy subgroups, we further conducted DAGs for these subgroups separately. In the DAG for the high schizotypy group (*n* = 452), SPQ-I had 2 edges pointing toward depressed mood and emotion suppression, and influenced stress and anxiety through depressed mood. The depressed mood had an edge pointing toward SPQ-D. In the DAG for the low schizotypy group (*n* = 482), depressed mood appeared to drive anxiety, and stress (see Supplementary Figure S1.11). Additionally, anxiety had an edge pointing toward SPQ-CP, with an arc strength of 82% (very close to the 85% threshold). In the sensitivity analysis for the high schizotypy subgroup, SPQ-I still had an edge pointing toward depressed mood with an arc strength of 83% (very close to the 85% threshold). Depressed mood still had an edge pointing to SPQ-D. In the sensitivity analysis for the low schizotypal subgroup, depressed mood still appeared to drive anxiety and stress, and anxiety still has an edge pointing toward SPQ-CP.

In the DAG for children aged 9–10, the depressed mood had an edge pointing toward SPQ-D, while stress pointing toward both SPQ-D and SPQ-CP. For children aged 11–12, the DAG showed that depressed mood had an edge pointing toward SPQ-D, anxiety pointing toward SPQ-CP, and both depressed mood and stress pointing toward SPQ-I (see Supplementary Figure S1.12). In the sensitivity analysis, we identified similar directional relationships, with negative affect mainly pointing to the dimensions of schizotypal traits rather than the other way around (see Supplementary Material 2, S2.4 for details).

## Discussions

This study revealed the inter-relationship between schizotypal traits, emotion regulation, and negative affect in a large sample of children. The key findings can be summarized as follows. In the regularized partial correlation network, all 3 dimensions of schizotypal traits were positively correlated with negative affect. Emotion suppression was positively correlated with all negative affect nodes, while emotion reappraisal was negatively correlated with depressed mood and stress. Additionally, we found positive correlations of emotion suppression with SPQ-I and SPQ-D nodes. Emotion reappraisal correlated positively with SPQ-CP, and negatively with SPQ-I. Stress was pivotal in the network, as revealed in the node centrality indices. The DAGs revealed that stress plays a role in driving schizotypal traits. Moreover, gender, age, and schizotypy level (high/low) appeared to have an impact on the network patterns.

We examined the relationship between schizotypal traits and negative affect. Positive correlations were found between all dimensions of schizotypal traits and negative affect after controlling other variables in the network, consistent with earlier findings in adolescents^10,65^ and adults.^11,66^ This indicates that negative affect and schizotypal traits have been closely related during childhood. Previous research in adults has found that SPQ-D appears to show a stronger association with negative affect.^18–20^ However, in our sample of children, SPQ-CP rather than other SPQ nodes was more related to anxiety. Such inconsistent results might be due to sampling differences, and the fact that previous studies did not categorize different types of NA. Future research recruits samples with different age ranges, and categorizes NA to further clarify the relationship between schizotypal dimensions and negative affect. Our explorative DAG findings indicated that stress and depressed mood in children appeared to drive schizotypal traits. Additionally, stress exacerbated schizotypal symptoms via depressed mood and anxiety, partially concurring with previous findings on the mediating role of negative affect in the relationship between stress and mental health.^30^ Previous research has indicated that negative affect may play a role in the development and exacerbation of schizotypal symptoms. From a developmental perspective, schizotypal traits in developing children may be insufficient to determine the development of psychosis,^21,67^ negative affect may serve as aggravating factors to synergize the impact of schizotypal traits.^15,67^ However, causality may also be bidirectional between negative affect and schizotypal traits.^21^ It is important to note that DAGs do not confirm causality,^45^ and their results should be further clarified in future longitudinal studies.

Regarding the role of emotion regulation, we found that greater use of suppression was associated with more depressed mood, anxiety, and stress, while greater use of emotion reappraisal was associated with less depressed mood and stress. These are consistent with previous findings on suppression^26,68^ and reappraisal^26,69^ in children, and further support that suppression is a maladaptive emotion regulation strategy, while reappraisal is adaptive.^26,69^ Regarding schizotypal traits and emotion regulation, we found that SPQ-I and SPQ-D were positively correlated with emotion suppression, and emotion reappraisal was correlated positively with SPQ-CP but negatively with SPQ-I. A study using experience sampling showed that individuals with psychosis and active delusions had used maladaptive strategies more often than healthy people, and had used reappraisal more frequently.^33^ Such findings concurred with ours. Having said that, we found that emotion reappraisal had weaker relationships with other nodes in the network, as evidenced by its significantly lowest EI centrality and bridge EI. As children and adolescents grow older, emotion reappraisal might become more frequent and effective.^70–72^

The extant literature generally suggested that reliability of closeness and betweenness would be poor,^73,74^ and strength and EI can be better indicators of central nodes. In our study, stress showed significantly higher strength than all other nodes, except depressed mood, and stress also showed the highest EI, suggesting that stress might play an important role in the network. Although some network analysis studies regarded high-centrality and bridge nodes as intervention targets,^38,39^ recent research advocated a more conservative approach to interpret these indices.^75,76^ Future research can further explore the roles of these central and bridge nodes to better understand their potential roles.

Our preliminary NCT results also explored the effects of age, gender, and schizotypy levels on the network. Although girls scored significantly higher than boys on SPQ, negative affect, emotion suppression, and reappraisal, the NCT results revealed no significant between-gender differences in the network, except that emotion reappraisal had higher EI in girls than boys. Such findings suggested that reappraisal might be more important for girls, consistently with the notion that girls generally have greater awareness and intention to regulate emotions than boys of the same age.^77^ However, prior research using children of similar age groups reported mixed results, regarding gender differences.^47,78–82^ Our findings should be considered preliminary and interpreted with caution. Furthermore, our findings suggested significant effects of age, because the network structure and global strength between the subgroups aged 9–10 and 11–12 differed significantly. Specifically, children aged 11–12 had a stronger connection between SPQ-CP and anxiety compared with those aged 9–10. The DAG results also revealed differences across different age groups. It is noteworthy that our findings remained preliminary. Future research could recruit samples with a wider age range, and utilize longitudinal designs to better understand developmental changes. Additionally, our findings supported that the high and low schizotypy subgroups differed significantly. For instance, the connection between SPQ-D and depressed mood was stronger in the high schizotypy group. The DAG analysis showed an effect of SPQ-I on depressed mood in the high schizotypy group, but not in the low schizotypy group. A previous network analysis in adults also found stronger correlations between schizotypal traits and negative affect in the high schizotypy group.^83^ Having said that, the use of NCT to explore the effects of age, gender, and high versus low schizotypy level was preliminary, and further research in this area is necessary.

This study has several limitations. First, we did not collect sufficient socioeconomic information, which might have confounded the findings. However, a recent study on Chinese children did not find any significant correlation between the ratings on the SPQ-C and demographics.^47^ Second, we did not include a lie scale to detect random or dishonest responses, thus undermined the validity of our findings. Third, we used self-report scales only, but many variables of interest, such as emotion regulation, can be measured objectively by laboratory-based paradigms. Fourth, our sample had a narrowed age range, which limited the generalizability. Fifth, we only measured emotion reappraisal and suppression, but other strategies such as rumination and acceptance may also be important in modifying psychopathology. Finally, network analyses have potential limitations, including problems of generalizability and reproducibility of network estimation.^76^ The centrality indices have been criticized for lacking a theoretical foundation.^76^ Targeting central and bridging symptoms based on a single time-point identification is not the ideal approach for network-guided interventions.^74^ DAGs with cross-sectional design precluded the possibility to infer causality, and our findings need to be verified in future longitudinal studies. HC algorithms may yield suboptimal networks and biased results, however, these issues can be partially mitigated with ad hoc computational strategies.^60^ In our study, some important variables (ie, childhood trauma) were not measured,^84,85^ limiting both the partial correlation and DAG-based analyses. Future research should consider incorporating these variables in the networks.

To conclude, this network analysis suggested that children with higher levels of schizotypal traits may experience more negative affect and are more likely to use suppression to regulate these emotions. The Bayesian network revealed that stress may serve as a conditional influential node for schizotypal traits. Our preliminary NCT findings suggested that age and schizotypy level (high/low) might influence the network properties. Our work paved the way for future longitudinal or experimental studies, to guide the identification of intervention targets and to understand the pathological mechanisms in children.

## Supplementary Material

Supplementary material is available at https://academic.oup.com/schizophreniabulletin/.

sbae172_suppl_Supplementary_Materials
