# Supplementary material for: Relationship Between Schizotypal Traits, Emotion Regulation, and Negative Affect in Children: A Network Analysis
Source: Schizophr Bull. 2025 Mar 4;51(Suppl 2):S226–37. doi: 10.1093/schbul/sbae172 (PMC11879503; doi:10.1093/schbul/sbae172)
Supplement: sbae172_suppl_Supplementary_Materials [file sbae172_suppl_supplementary_materials.zip › Supplementary Materials_1 13_08_24.docx]

**Supplementary Materials_1**

**Relationship between schizotypal traits, emotion regulation and negative affect in children: A network analysis**

**Analyses in the maintext**

**Supplementary Materials_1**

**S1.1. Handling Missing Values**

**Figure S1.1** Visualiation missing values in each variable.

**S1.2. Network Analysis Results for the Whole Sample**

**S1.2.1** Supplementary Results for Whole-Sample Network Analysis

**Table S1.1** Centrality, predictability, and expected influence of nodes in the whole-sample network

**Table S1.2** Edge-weights in the whole-sample network

**Table S1.3** Bivariate correlations of all measurements and their subscales

**Figure S1.2** Bootstrapped difference tests between edge-weights in the network of whole sample

**Figure S1.3** Bootstrapped difference test for node strength centrality in the network of whole sample

**S1.2.2** Supplementary Results for Bridge Centrality

**Figure S1.4** The standardized bridge centrality estimates of the whole-sample network.

**Figure S1.5** Bootstrapped difference test for bridge centrality indices

**Figure S1.6** The stability of bridge centrality indicators computed by case-dropping bootstrap

**S1.3 Gender, Age, and High/Low Group Differences Analysis**

**S1.3.1** Figures for Network Comparisons

**Figure S1.7** Regularized partial networks in boys and girls

**Figure S1.8** Regularized partial networks in children aged 9-10 years and 11-12 years

**Figure S1.9** Regularized partial networks in low schizotypy and high schizotypy groups

**S1.3.2** Tables for Independent sample t tests

**Table S1.4** Gender difference in age, schizotypal traits, emotion regulation and negative affect

**Table S1.5** Age-group difference in schizotypal traits, emotion regulation and negative affect

**Table S1.6** High/Low schizotypy difference in age, schizotypal traits, emotion regulation and negative affect

**S1.4 Results for Bayesian network**

**S1.3.1 Figures for Bayesian networks**

**Figure S1.10** A Bayesian network for the whole sample

**Figure S1.11** Estimated Bayesian networks for the low and high schizotypy groups

**Figure S1.12** Estimated Bayesian networks in children aged 9-10 years and 11-12 years

**S1.3.2 Tables for Bayesian networks**

**Table S1.7** Arc strength estimated from the Bayesian networks of the whole sample **Table S1.8** Arc strength estimated from the Bayesian networks of the high schizotypy group

**Table S1.9** Arc strength estimated from the Bayesian networks of the low schizotypy group

**Table S1.10** Arc strength estimated from the Bayesian networks of the 9-10 age group

**Table S1.11** Arc strength estimated from the Bayesian networks of the 11-12 age group

**S1.1. Handling Missing Values**

**Handling with missing values**

In the present study, a total of 1333 participants were initially included. 295 participants were excluded due to any missing values in the main variables, including scale items, age, and gender. We used the naniar package in R to analyze missing value patterns. The Figure S1 provided in the supplementary materials is to visually illustrate the missingness.

We replaced the data in the dataset with indicator variables (1 representing missing, 0 representing present), resulting in a shadow matrix. Computing the correlations among these indicator variables can help to identify which two variables have missing simultaneously. Computing the correlations among these indicator variables and the original variables can help analyze what variables are related to missing data. The correlation coefficients between missing values and other variables range from -0.08 to 0.16 (correlation between Dass7 and Dass15_missing), indicating weak correlations between missing values and the original variables.

Therefore, the missing values in this study can be considered as randomly generated and do not have any special correlation with the variables of interest in this study.

| (A)   | (B)   |
| --- | --- |
| **Figure S1.1** (A) Visualiation missing values in each variable. (B) Visualization of missing data, the locations of missing values are depicted in black, and the overall percentage of missing values displayed in the legend. | |

**S1.2. Network Analysis Results for the Whole Sample**

**S1.2.1 Supplementary Results for Whole-Sample Network Analysis**

**Table S1.1 Centrality, predictability, and expected influence of nodes in the whole-sample network**

|  | Strength | Closeness | Betweenness | Expected Influence | Predictability |
| --- | --- | --- | --- | --- | --- |
| Cognitive perceptual | -0.02 | 0.18 | 0.55 | 0.37 | 0.49 |
| Interpersonal | -0.09 | 0.63 | 0.06 | -0.05 | 0.48 |
| Disorganized | -1.45 | -1.11 | -0.92 | -0.72 | 0.42 |
| Reappraisal | -1.07 | -1.34 | -0.92 | -1.97 | 0.26 |
| Suppression | -0.33 | -0.95 | 0.06 | 0.13 | 0.37 |
| depression | 1.01 | 1.33 | 2.03 | 0.17 | 0.64 |
| Anxiety | 0.39 | 0.45 | 0.06 | 0.68 | 0.66 |
| Stress | 1.57 | 0.82 | -0.92 | 1.40 | 0.69 |

**Table S1.2 Edge-weights in the whole-sample network**

|  | SPQ-CP | SPQ-I | SPQ-D | ERQ-R | ERQ-S | DASS-D | DASS-A | DASS-S |
| --- | --- | --- | --- | --- | --- | --- | --- | --- |
| SPQ-CP | 0.00 |  |  |  |  |  |  |  |
| SPQ-I | 0.31 | 0.00 |  |  |  |  |  |  |
| SPQ-D | 0.19 | 0.09 | 0.00 |  |  |  |  |  |
| ERQ-R | 0.05 | -0.04 | 0.00 | 0.00 |  |  |  |  |
| ERQ-S | 0.00 | 0.11 | 0.03 | 0.48 | 0.00 |  |  |  |
| DASS-D | 0.04 | 0.16 | 0.12 | -0.12 | 0.13 | 0.00 |  |  |
| DASS-A | 0.20 | 0.02 | 0.09 | 0.00 | 0.01 | 0.24 | 0.00 |  |
| DASS-S | 0.12 | 0.15 | 0.12 | -0.02 | 0.09 | 0.28 | 0.40 | 0.00 |

Note:SPQ-CP = cognitive perceptual dimension of Schizotypal Personality Questionnaire; SPQ-I = interpersonal dimension of Schizotypal Personality Questionnaire; SPQ-D = disorganized dimension of Schizotypal Personality Questionnaire; ERQ-R= reappraisal dimension of Emotion Regulation Questionnaire; ERQ-S= suppression dimension of Emotion Regulation Questionnaire; DASS-D= depression dimension of Depression Anxiety Stress Scales; DASS-A= anxiety dimension of Depression Anxiety Stress Scales; DASS-S= stress dimension of Depression Anxiety Stress Scales;

**Table S1.3 Bivariate correlations of all measurements and their subscales**

|  | SPQ-total | SPQ-CP | SPQ-I | SPQ-D | ERQ- total | ERQ-R | ERQ-S | DASS-total | DASS-D | DASS-A | DASS-S |
| --- | --- | --- | --- | --- | --- | --- | --- | --- | --- | --- | --- |
| SPQ-total | 1.00 |  |  |  |  |  |  |  |  |  |  |
| SPQ-CP | 0.89** | 1.00** |  |  |  |  |  |  |  |  |  |
| SPQ-I | 0.87** | 0.62** | 1.00 |  |  |  |  |  |  |  |  |
| SPQ-D | 0.72** | 0.54** | 0.49** | 1.00 |  |  |  |  |  |  |  |
| ERQ- total | 0.20** | 0.18** | 0.17** | 0.14** | 1.00 |  |  |  |  |  |  |
| ERQ-R | 0.06* | 0.08** | 0.03 | 0.04 | 0.91** | 1.00 |  |  |  |  |  |
| ERQ-S | 0.36** | 0.29** | 0.34** | 0.27** | 0.77** | 0.46** | 1.00 |  |  |  |  |
| DASS-total | 0.77** | 0.67** | 0.65** | 0.60** | 0.18** | 0.03 | 0.38** | 1.00 |  |  |  |
| DASS-D | 0.67** | 0.56** | 0.58** | 0.52** | 0.14** | -0.02 | 0.35** | 0.85** | 1.00 |  |  |
| DASS-A | 0.68** | 0.62** | 0.56** | 0.53** | 0.17** | 0.05 | 0.32** | 0.91** | 0.68** | 1.00 |  |
| DASS-S | 0.71** | 0.62** | 0.61** | 0.56** | 0.18** | 0.03 | 0.36** | 0.92** | 0.71** | 0.75** | 1.00 |

Note: SPQ-total = total scores of Schizotypal Personality Questionnaire; ERQ-total = total scores of Emotion Regulation Questionnaire; DASS-total = total score of Depression Anxiety Stress Scales; SPQ-CP = cognitive perceptual dimension of Schizotypal Personality Questionnaire; SPQ-I = interpersonal dimension of Schizotypal Personality Questionnaire; SPQ-D = disorganized dimension of Schizotypal Personality Questionnaire; ERQ-R= reappraisal dimension of Emotion Regulation Questionnaire; ERQ-S= suppression dimension of Emotion Regulation Questionnaire; DASS-D= depression dimension of Depression Anxiety Stress Scales; DASS-A= anxiety dimension of Depression Anxiety Stress Scales; DASS-S= stress dimension of Depression Anxiety Stress Scales;

| **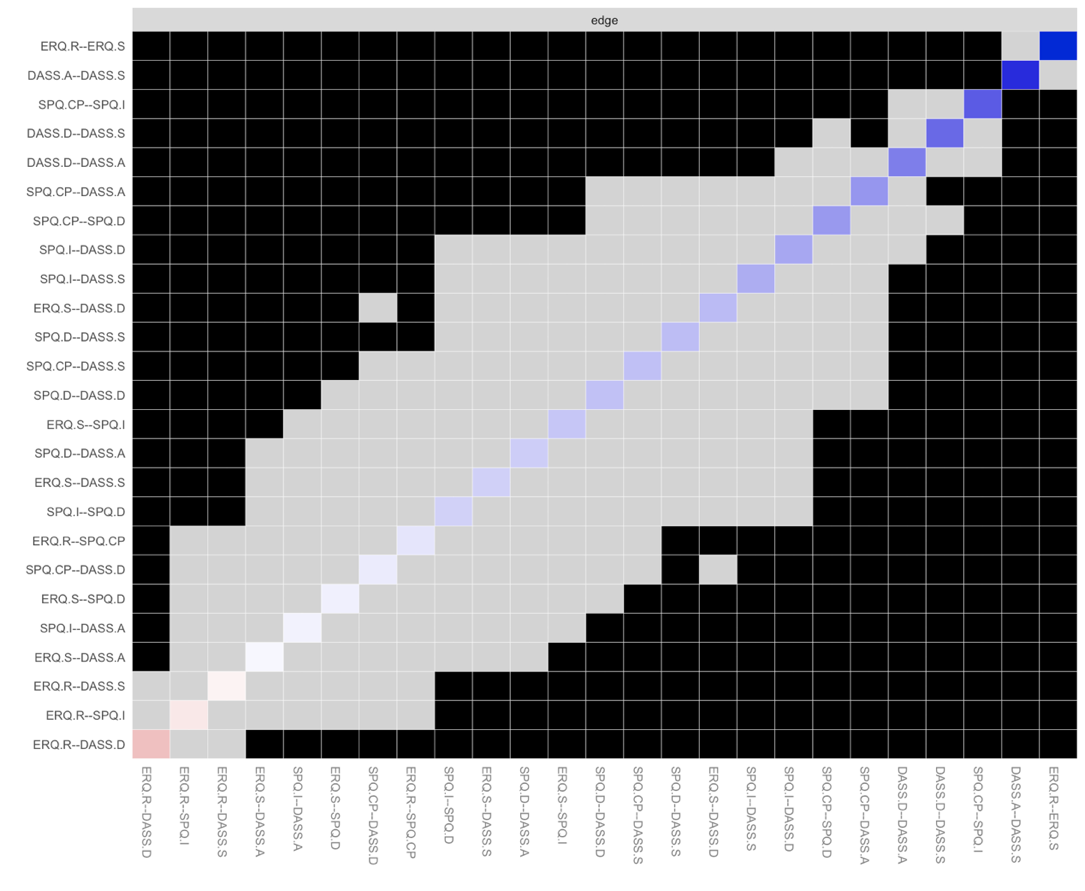** |
| --- |
| **Figure S1.2 Bootstrapped difference tests between edge-weights in the network of whole sample**  Note: Black boxes show edges that differed significantly form one another edges, and grey boxes show edges that were not significantly different. SPQ.CP = cognitive perceptual dimension of Schizotypal Personality Questionnaire; SPQ.I = interpersonal dimension of Schizotypal Personality Questionnaire; SPQ.D = disorganized dimension of Schizotypal Personality Questionnaire; ERQ.R= reappraisal dimension of Emotion Regulation Questionnaire; ERQ.S= suppression dimension of Emotion Regulation Questionnaire; DASS.D= depression dimension of Depression Anxiety Stress Scales; DASS.A= anxiety dimension of Depression Anxiety Stress Scales; DASS.S= stress dimension of Depression Anxiety Stress Scales; |

| **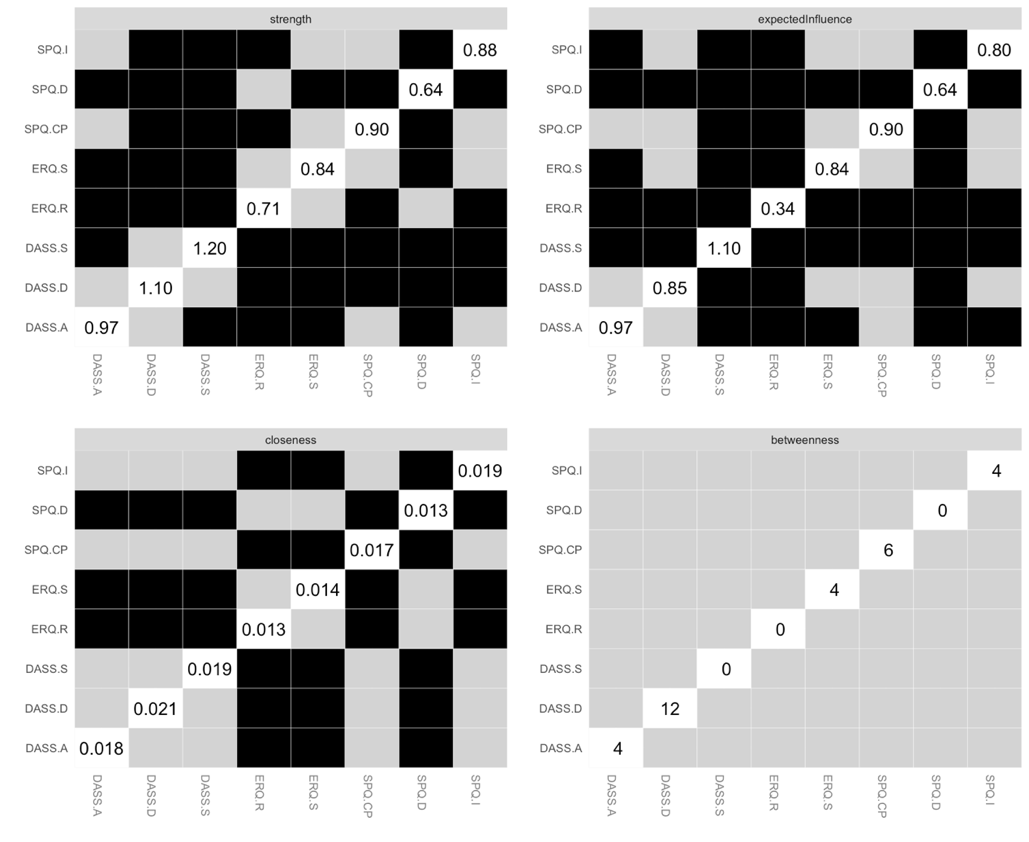** |
| --- |
| **Figure S1.3 Bootstrapped difference test for node centrality indices (from left to right and top to bottom: strength, EI, closeness, betweenness) in the network of whole sample**  Note: Black boxes show strengths of nodes that differed significantly form one another, while grey boxes shows strength of nodes that were not significantly different. SPQ.CP = cognitive perceptual dimension of Schizotypal Personality Questionnaire; SPQ.I = interpersonal dimension of Schizotypal Personality Questionnaire; SPQ.D = disorganized dimension of Schizotypal Personality Questionnaire; ERQ.R= reappraisal dimension of Emotion Regulation Questionnaire; ERQ.S= suppression dimension of Emotion Regulation Questionnaire; DASS.D= depression dimension of Depression Anxiety Stress Scales; DASS.A= anxiety dimension of Depression Anxiety Stress Scales; DASS.S= stress dimension of Depression Anxiety Stress Scales; |

**S1.2.2 Supplementary Results for Bridge Centrality**

| 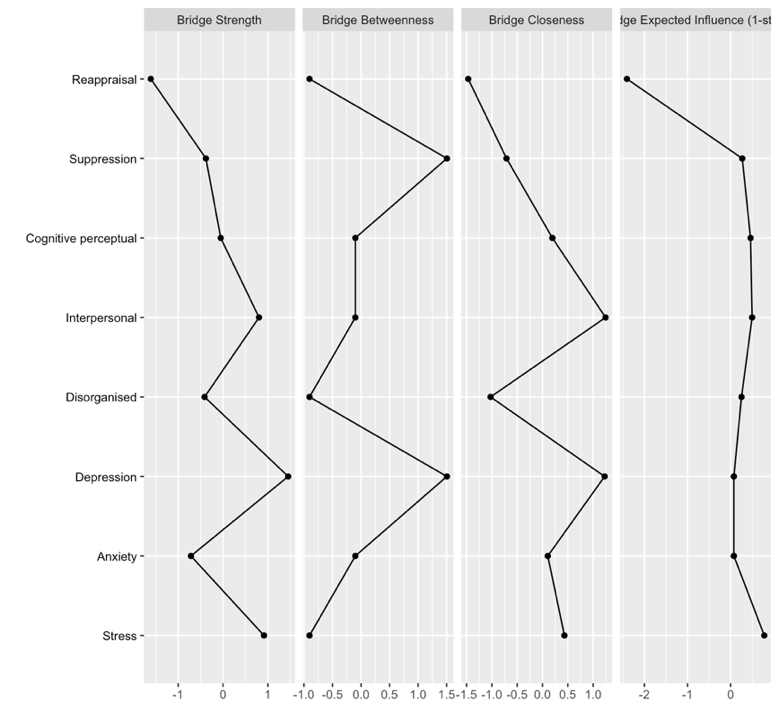 |
| --- |
| **Figure S1.4 The standardized bridge centrality estimates of the whole-sample network.** |

| 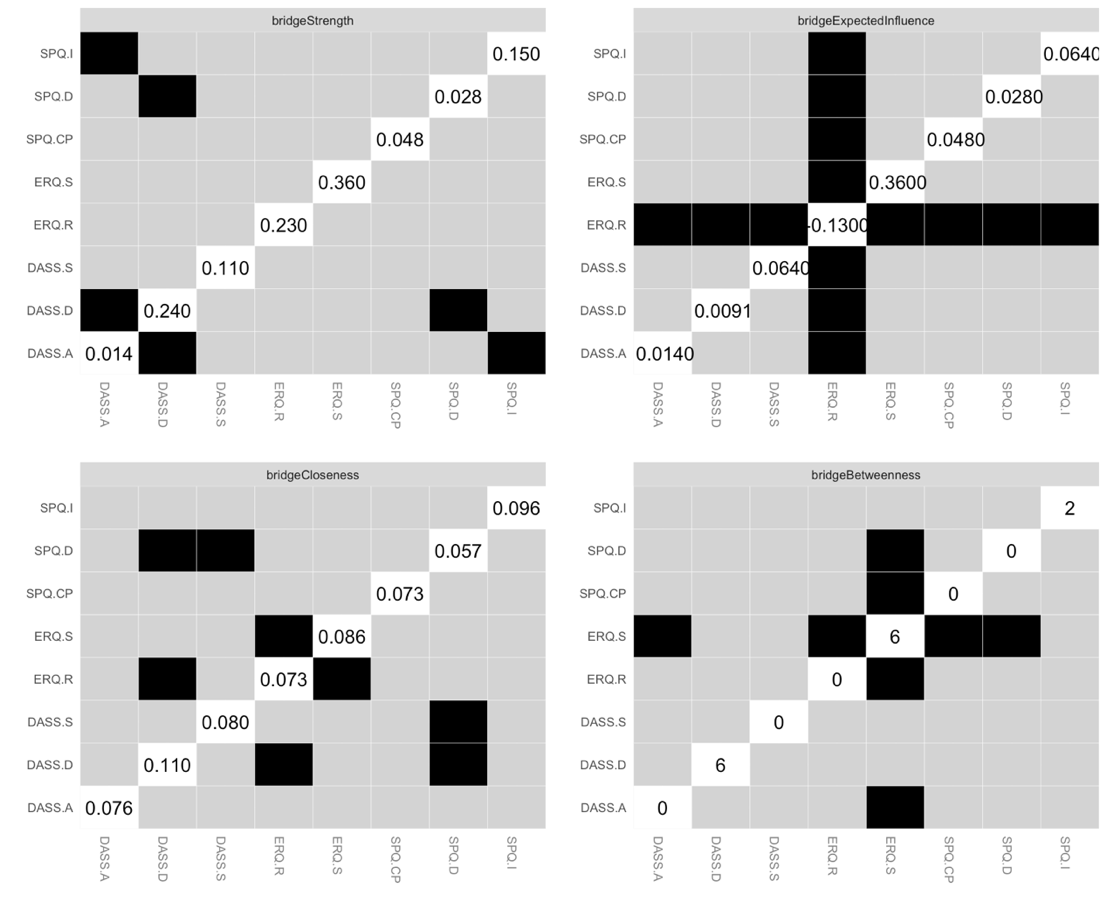 |
| --- |
| **Figure S1.5 Bootstrapped difference test for bridge centrality indices (from left to right and top to bottom: bridgeStrength, bridgeEI, bridgeCloseness, bridgeBetweenness) in the network of whole sample**  Note: Black boxes shows strengths of nodes that differed significantly form one another, while grey boxes shows strength of nodes that were not significantly different. SPQ.CP = cognitive perceptual dimension of Schizotypal Personality Questionnaire; SPQ.I = interpersonal dimension of Schizotypal Personality Questionnaire; SPQ.D = disorganized dimension of Schizotypal Personality Questionnaire; ERQ.R= reappraisal dimension of Emotion Regulation Questionnaire; ERQ.S= suppression dimension of Emotion Regulation Questionnaire; DASS.D= depression dimension of Depression Anxiety Stress Scales; DASS.A= anxiety dimension of Depression Anxiety Stress Scales; DASS.S= stress dimension of Depression Anxiety Stress Scales; |

| 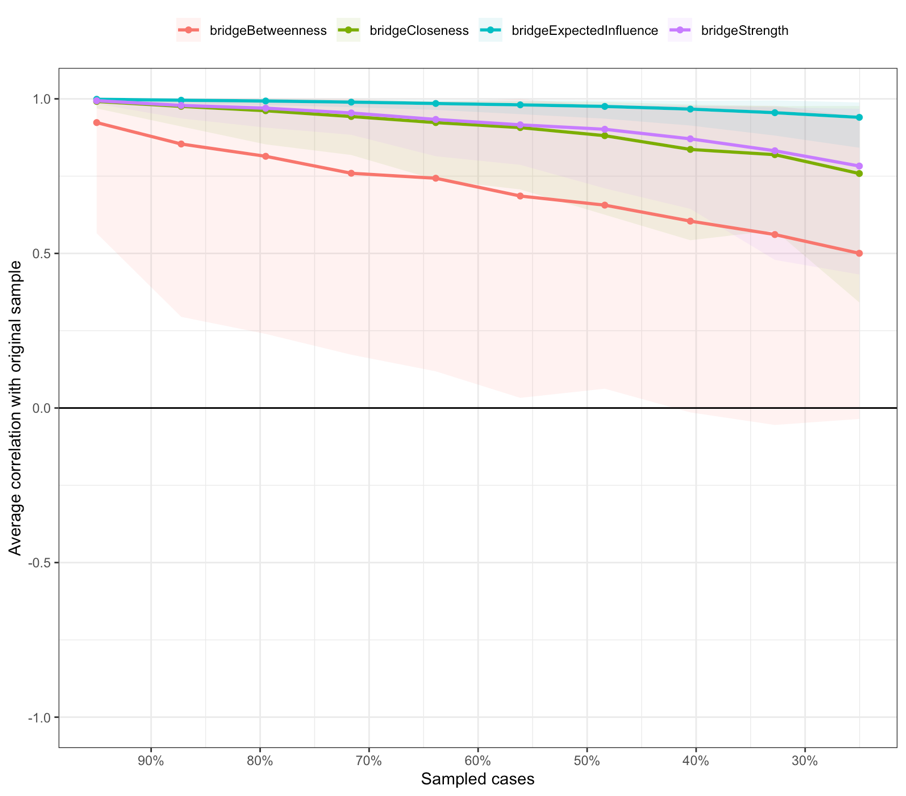 |
| --- |
| **Figure S1.6 The stability of bridge centrality indicators computed by case-dropping bootstrap.**  Note: The lines represent the average correlation between the central indicators estimated from the bootstrapping subsamples and the bridge central indicators estimated from the original sample. The areas represent a range from 2.5th quantiles to 97.5th quantiles. |

**S1.3 Gender, Age, and High/Low Group Differences Analysis**

**S1.3.1 Figures for Network Comparisons**

| 1. Boys   **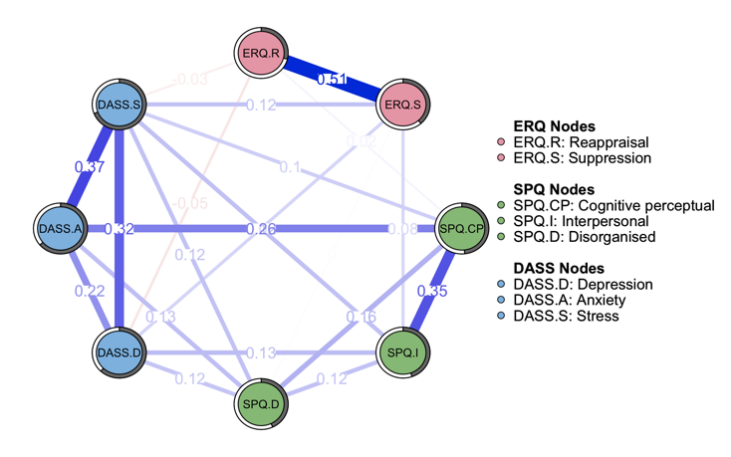** | 1. Girls   **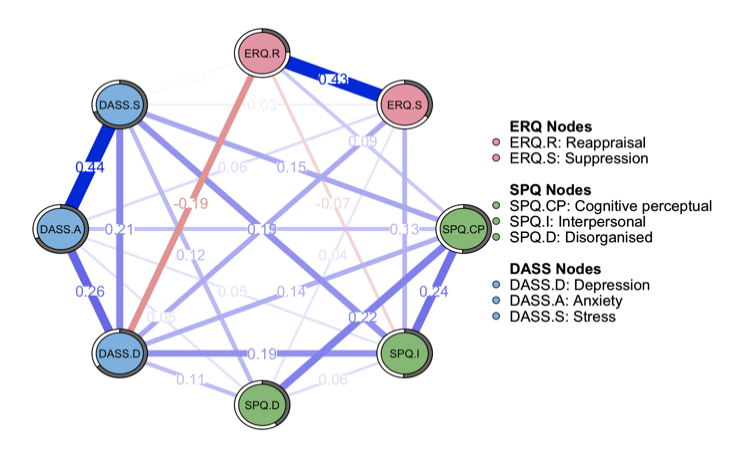** |
| --- | --- |
| **Figure S1.7 Regularized partial networks in boys (left, n=540) and girls (right, n=479)**  Each node represents a variable. Each edge represents the partial correlation between two nodes controlled for all other nodes. Thicker lines represent stronger connections. The value of each edge represents the strength of the partial correlations. The blue lines indicate positive partial correlations and the red lines indicate negative partial correlations. The blue ring around each node represents the predictability values, which indicates prediction of a specific node by other nodes in the network.  Note: SPQ.CP = cognitive perceptual dimension of Schizotypal Personality Questionnaire; SPQ.I = interpersonal dimension of Schizotypal Personality Questionnaire; SPQ.D = disorganized dimension of Schizotypal Personality Questionnaire; ERQ.R= reappraisal dimension of Emotion Regulation Questionnaire; ERQ.S= suppression dimension of Emotion Regulation Questionnaire; DASS.D= depression dimension of Depression Anxiety Stress Scales; DASS.A= anxiety dimension of Depression Anxiety Stress Scales; DASS.S= stress dimension of Depression Anxiety Stress Scales; | |

| (A) 9-11 age group  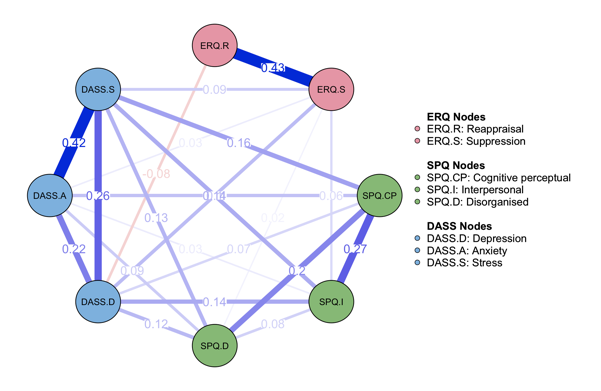 | (B) 11-12 age group  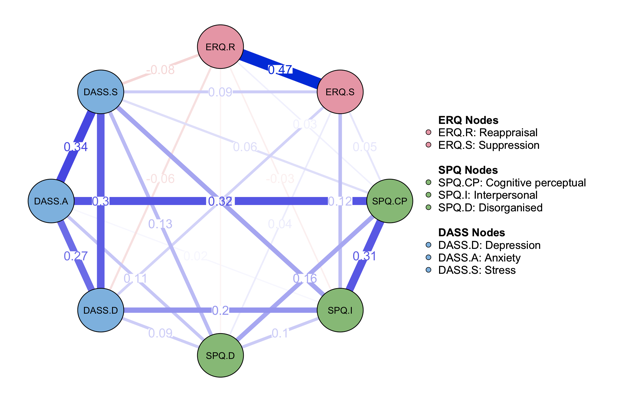 |
| --- | --- |
| **Figure S1.8 Regularized partial networks in children aged 9-10 years (left, n=544) and 11-12 years (right, n=475)**  Each node represents a variable. Each edge represents the partial correlation between two nodes controlled for all other nodes. Thicker lines represent stronger connections. The value of each edge represents the strength of the partial correlations. The blue lines indicate positive partial correlations and the red lines indicate negative partial correlations. The blue ring around each node represents the predictability values, which indicates prediction of a specific node by other nodes in the network.  Note: SPQ.CP = cognitive perceptual dimension of Schizotypal Personality Questionnaire; SPQ.I = interpersonal dimension of Schizotypal Personality Questionnaire; SPQ.D = disorganized dimension of Schizotypal Personality Questionnaire; ERQ.R= reappraisal dimension of Emotion Regulation Questionnaire; ERQ.S= suppression dimension of Emotion Regulation Questionnaire; DASS.D= depression dimension of Depression Anxiety Stress Scales; DASS.A= anxiety dimension of Depression Anxiety Stress Scales; DASS.S= stress dimension of Depression Anxiety Stress Scales; | |

| (A) Low schizotypy  **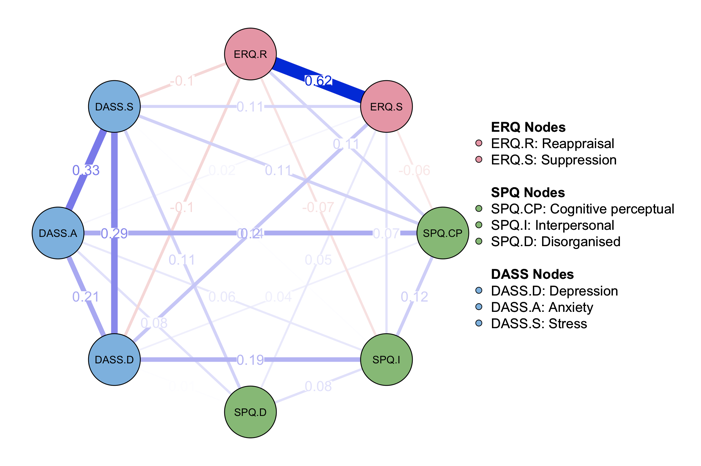** | (B) High schizotypy  **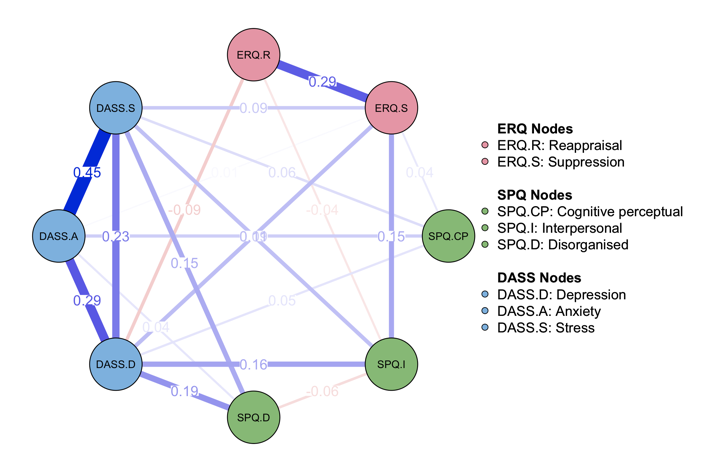** |
| --- | --- |
| **Figure S1.9 Regularized partial networks in low schizotypy (left, n=482) and high schizotypy (right, n=452) groups.**  Each node represents a variable. Each edge represents the partial correlation between two nodes controlled for all other nodes. Thicker lines represent stronger connections. The value of each edge represents the strength of the partial correlations. The blue lines indicate positive partial correlations and the red lines indicate negative partial correlations. The blue ring around each node represents the predictability values, which indicates prediction of a specific node by other nodes in the network.  Note: SPQ.CP = cognitive perceptual dimension of Schizotypal Personality Questionnaire; SPQ.I = interpersonal dimension of Schizotypal Personality Questionnaire; SPQ.D = disorganized dimension of Schizotypal Personality Questionnaire; ERQ.R= reappraisal dimension of Emotion Regulation Questionnaire; ERQ.S= suppression dimension of Emotion Regulation Questionnaire; DASS.D= depression dimension of Depression Anxiety Stress Scales; DASS.A= anxiety dimension of Depression Anxiety Stress Scales; DASS.S= stress dimension of Depression Anxiety Stress Scales; | |

**S1.3.2 Tables for Independent sample t tests**

**Table S1.4** *Gender difference in age, schizotypal traits, emotion regulation and negative affect*

|  | Sample  (Mean ± standard deviation) | | *t* | *df* | *p* | *Cohen's d* |
| --- | --- | --- | --- | --- | --- | --- |
|  | Boys  (n=540) | Girls  (n=479) |  |  |  |  |
| Age (years) | 10.36±0.97 | 10.43±0.94 | -1.25 | 1017 | 0.21 | -0.08 |
| Length of education (years) | 4.94±0.83 | 5.07±0.81 | -2.50 | 1017 | 0.01 | -0.16 |
|  |  |  |  |  |  |  |
| SPQ-C total score | 5.83±4.45 | 6.79±4.75 | -3.32 | 1017 | 0.001 | -0.21 |
| SPQ-C cognitive perceptual | 2.69±2.07 | 3.00±2.08 | -2.37 | 1017 | 0.02 | -0.15 |
| SPQ-C interpersonal | 2.17±1.90 | 2.64±2.21 | -3.65 | 949.86 | <.001 | -0.23 |
| SPQ-C disorganized | 0.97±1.38 | 1.15±1.36 | -2.03 | 1017 | 0.04 | -0.13 |
|  |  |  |  |  |  |  |
| ERQ-CA total score | 27.69±9.93 | 29.67±9.03 | -3.34 | 1016.38 | 0.001 | -0.21 |
| ERQ-CA suppression | 9.71±4.25 | 10.94±4.31 | -4.58 | 1017 | <.001 | -0.29 |
| ERQ-CA cognitive reappraisal | 17.98±6.99 | 18.73±6.39 | -1.81 | 1016.05 | 0.07 | -0.11 |
|  |  |  |  |  |  |  |
| DASS-21 total score | 9.64±10.13 | 12.45±11.63 | -4.09 | 954.40 | <.001 | -0.26 |
| DASS-21 depression | 2.48±3.50 | 3.43±4.24 | -3.88 | 929.60 | <.001 | -0.25 |
| DASS-21 anxiety | 3.66±3.86 | 4.63±4.26 | -3.82 | 971.50 | <.001 | -0.24 |
| DASS-21 stress | 3.50±3.83 | 4.38±4.24 | -3.47 | 969.45 | 0.001 | -0.22 |

Note: SPQ-C: Schizotypal Personality Questionnaire- Children; ERQ-CA: Emotion Regulation. Questionnaire for Children and Adolescent; DASS-21: The Depression Anxiety Stress Scales.If Levene's test indicated that the variances were equal across the two groups (p>0.05), we reported the results of the t-test assuming equal variances. Conversely, if Levene's test indicated that the variances were not equal across the two groups (p<0.05), we reported the results of the t-test assuming unequal variances.

**Table S1.5** *Age-group difference in schizotypal traits, emotion regulation and negative affect*

|  | Sample  (Mean ± standard deviation) | | *t* | *df* | *p* | *Cohen's d* |
| --- | --- | --- | --- | --- | --- | --- |
|  | 9-10  (n=544) | 11-12  (n=475) |  |  |  |  |
| Age (years) | 9.62±0.49 | 11.28±0.45 | -56.69 | 1013.19 | <.001 | -3.54 |
| Length of education (years) | 4.38±0.49 | 5.72±0.47 | -44.36 | 1005.14 | <.001 | -2.78 |
|  |  |  |  |  |  |  |
| SPQ-C total score | 6.19±4.32 | 6.38±4.94 | -0.65 | 948.89 | 0.52 | -0.04 |
| SPQ-C cognitive perceptual | 2.86±2.04 | 2.80±2.13 | 0.46 | 1017 | 0.64 | 0.03 |
| SPQ-C interpersonal | 2.36±1.96 | 2.42±2.18 | -0.45 | 960.68 | 0.65 | -0.03 |
| SPQ-C disorganized | 0.97±1.30 | 1.16±1.45 | -2.20 | 958.43 | **0.03** | -0.14 |
|  |  |  |  |  |  |  |
| ERQ-CA total score | 28.95±9.55 | 28.24±9.58 | 1.19 | 1017 | 0.24 | 0.07 |
| ERQ-CA suppression | 10.31±4.29 | 10.25±4.35 | 0.22 | 1017 | 0.83 | 0.01 |
| ERQ-CA cognitive reappraisal | 18.64±6.71 | 17.99±6.72 | 1.55 | 1017 | 0.12 | 0.10 |
|  |  |  |  |  |  |  |
| DASS-21 total score | 10.62±10.49 | 11.34±11.45 | -1.04 | 969.14 | 0.30 | -0.07 |
| DASS-21 depression | 2.61±3.46 | 3.30±4.31 | -2.80 | 906.25 | **0.01** | -0.18 |
| DASS-21 anxiety | 4.13±4.16 | 4.09±3.99 | 0.15 | 1017 | 0.88 | 0.01 |
| DASS-21 stress | 3.88±3.95 | 3.95±4.17 | -0.25 | 1017 | 0.80 | -0.02 |

Note: SPQ-C: Schizotypal Personality Questionnaire- Children; ERQ-CA: Emotion Regulation. Questionnaire for Children and Adolescent; DASS-21: The Depression Anxiety Stress Scales.If Levene's test indicated that the variances were equal across the two groups (p>0.05), we reported the results of the t-test assuming equal variances. Conversely, if Levene's test indicated that the variances were not equal across the two groups (p<0.05), we reported the results of the t-test assuming unequal variances.

**Table S1.6** *High/Low schizotypy difference in age, schizotypal traits, emotion regulation and negative affect*

|  | Sample  (Mean ± standard deviation) | | *t* | *df* | *p* | *Cohen's d* |
| --- | --- | --- | --- | --- | --- | --- |
|  | Low  (n=482) | High  (n=452) |  |  |  |  |
| Age (years) | 10.41±0.91 | 10.40±0.99 | 0.17 | 913.18 | 0.87 | 0.01 |
| Length of education (years) | 5.01±0.81 | 5.03±0.83 | -0.38 | 932 | 0.70 | -0.03 |
|  |  |  |  |  |  |  |
| SPQ-C total score | 2.27±1.75 | 10.60±2.99 | -51.61 | 717.65 | <.001 | -3.43 |
| SPQ-C cognitive perceptual | 1.19±1.11 | 4.58±1.51 | -38.79 | 825.28 | <.001 | -2.56 |
| SPQ-C interpersonal | 0.85±1.06 | 4.05±1.69 | -34.50 | 750.42 | <.001 | -2.29 |
| SPQ-C disorganized | 0.23±0.50 | 1.98±1.51 | -23.33 | 541.34 | <.001 | -1.57 |
|  |  |  |  |  |  |  |
| ERQ-CA total score | 26.89±10.55 | 30.54±8.06 | -5.95 | 896.10 | <.001 | -0.39 |
| ERQ-CA suppression | 9.02±4.27 | 11.62±4.00 | -9.58 | 932 | <.001 | -0.63 |
| ERQ-CA cognitive reappraisal | 17.87±7.42 | 18.92±5.86 | -2.40 | 905.81 | 0.02 | -0.16 |
|  |  |  |  |  |  |  |
| DASS-21 total score | 4.26±5.33 | 18.32±11.39 | -23.90 | 630.10 | <.001 | -1.60 |
| DASS-21 depression | 0.95±1.72 | 5.18±4.52 | -18.68 | 571.52 | <.001 | -1.25 |
| DASS-21 anxiety | 1.78±2.24 | 6.65±4.30 | -21.53 | 668.28 | <.001 | -1.44 |
| DASS-21 stress | 1.54±2.23 | 6.49±4.20 | -22.30 | 675.45 | <.001 | -1.49 |

Note: SPQ-C: Schizotypal Personality Questionnaire- Children; ERQ-CA: Emotion Regulation. Questionnaire for Children and Adolescent; DASS-21: The Depression Anxiety Stress Scales.If Levene's test indicated that the variances were equal across the two groups (p>0.05), we reported the results of the t-test assuming equal variances. Conversely, if Levene's test indicated that the variances were not equal across the two groups (p<0.05), we reported the results of the t-test assuming unequal variances.

**S1.4 Results for Bayesian network**

**S1.3.1 Figures for Bayesian networks**

**
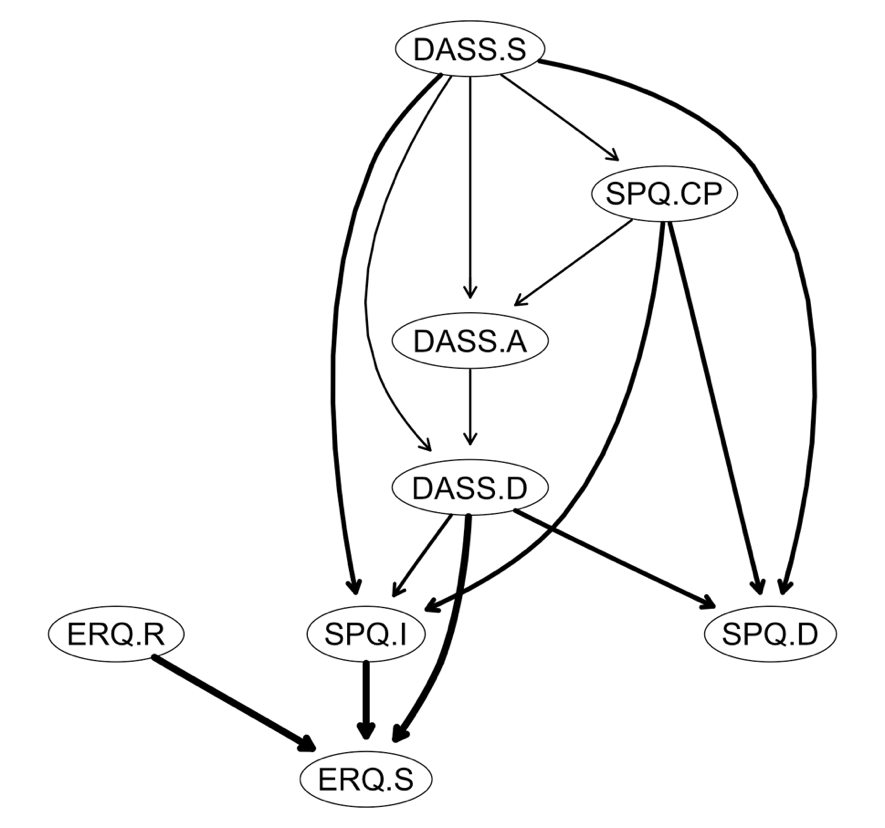
**

**Figure S1.10 A Bayesian network for the whole sample (directed acyclic graph; DAG)**

Note: The edge width corresponds to the direction probabilities, with thicker edges indicating higher direction probabilities. Only edges with probabilities exceeding 85% are displayed in the figure. SPQ.CP = cognitive perceptual dimension of Schizotypal Personality Questionnaire; SPQ.I = interpersonal dimension of Schizotypal Personality Questionnaire; SPQ.D = disorganized dimension of Schizotypal Personality Questionnaire; ERQ.R= reappraisal dimension of Emotion Regulation Questionnaire; ERQ.S= suppression dimension of Emotion Regulation Questionnaire; DASS.D= depression dimension of Depression Anxiety Stress Scales; DASS.A= anxiety dimension of Depression Anxiety Stress Scales; DASS.S= stress dimension of Depression Anxiety Stress Scales;

| (A) Low schizotypy  **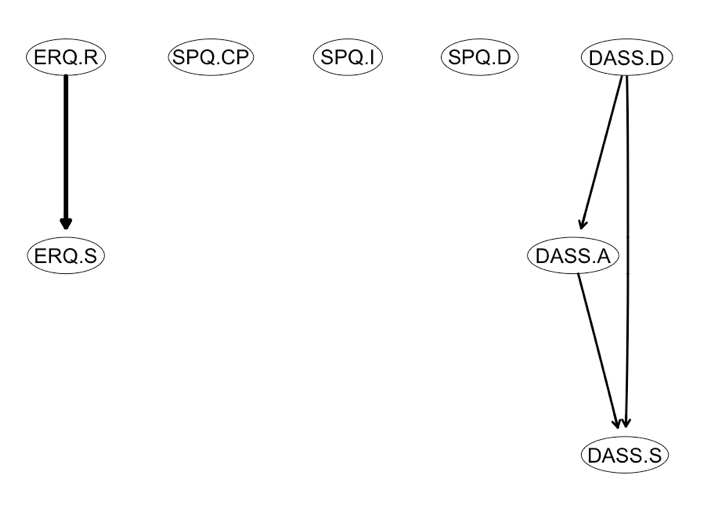** | (B) High schizotypy  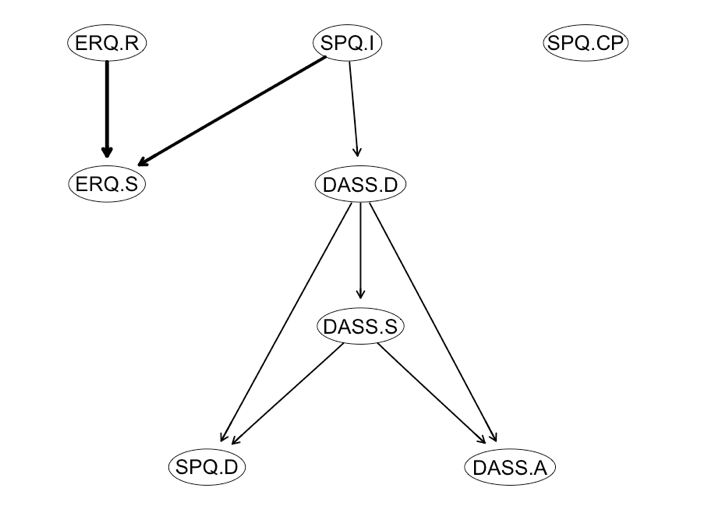 |
| --- | --- |
| **Figure S1.11 Estimated Bayesian networks for the low (left, n=482) and high (right, n=452) schizotypy groups.**  Note: The edge width corresponds to the direction probabilities, with thicker edges indicating higher direction probabilities. Only edges with probabilities exceeding 85% are displayed in the figure. SPQ.CP = cognitive perceptual dimension of Schizotypal Personality Questionnaire; SPQ.I = interpersonal dimension of Schizotypal Personality Questionnaire; SPQ.D = disorganized dimension of Schizotypal Personality Questionnaire; ERQ.R= reappraisal dimension of Emotion Regulation Questionnaire; ERQ.S= suppression dimension of Emotion Regulation Questionnaire; DASS.D= depression dimension of Depression Anxiety Stress Scales; DASS.A= anxiety dimension of Depression Anxiety Stress Scales; DASS.S= stress dimension of Depression Anxiety Stress Scales; | |

| (A) 9-10 age group  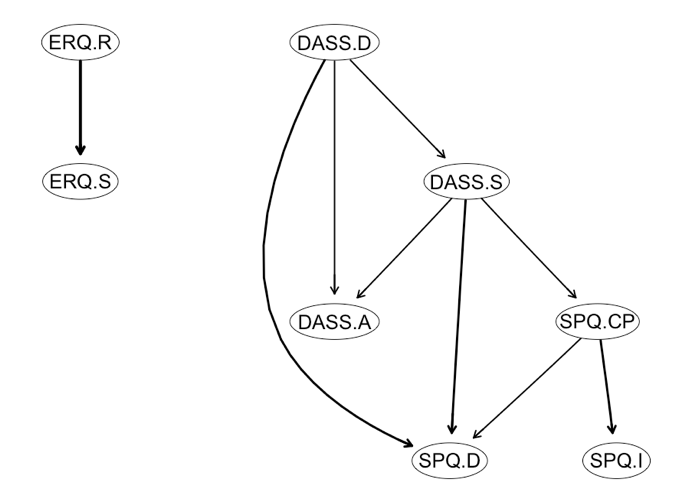 | (B) 11-12 age group  **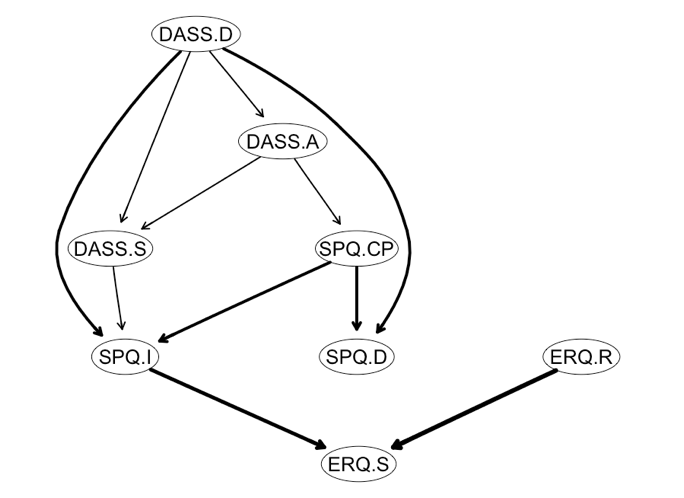** |
| --- | --- |
| **Figure S1.12 Estimated Bayesian networks in children aged 9-10 years (left, n=544) and 11-12 years (right, n=475)**  Note: The edge width corresponds to the direction probabilities, with thicker edges indicating higher direction probabilities. Only edges with probabilities exceeding 85% are displayed in the figure. SPQ.CP = cognitive perceptual dimension of Schizotypal Personality Questionnaire; SPQ.I = interpersonal dimension of Schizotypal Personality Questionnaire; SPQ.D = disorganized dimension of Schizotypal Personality Questionnaire; ERQ.R= reappraisal dimension of Emotion Regulation Questionnaire; ERQ.S= suppression dimension of Emotion Regulation Questionnaire; DASS.D= depression dimension of Depression Anxiety Stress Scales; DASS.A= anxiety dimension of Depression Anxiety Stress Scales; DASS.S= stress dimension of Depression Anxiety Stress Scales; | |

**S1.3.2 Tables for Bayesian networks**

**Table S1.7 Arc strength estimated from the Bayesian networks of the whole sample**

| From | To | Strength | Direction |
| --- | --- | --- | --- |
| ERQ.R | ERQ.S | 1.00 | 0.97 |
| SPQ.CP | SPQ.I | 1.00 | 0.76 |
| SPQ.CP | SPQ.D | 1.00 | 0.81 |
| SPQ.CP | DASS.A | 1.00 | 0.51 |
| SPQ.I | ERQ.S | 0.99 | 0.97 |
| DASS.D | ERQ.S | 0.92 | 0.95 |
| DASS.D | SPQ.I | 1.00 | 0.70 |
| DASS.D | SPQ.D | 1.00 | 0.81 |
| DASS.A | DASS.D | 1.00 | 0.56 |
| DASS.S | ERQ.S | 0.51 | 0.93 |
| DASS.S | SPQ.CP | 0.96 | 0.55 |
| DASS.S | SPQ.I | 0.95 | 0.76 |
| DASS.S | SPQ.D | 0.95 | 0.83 |
| DASS.S | DASS.D | 1.00 | 0.60 |
| DASS.S | DASS.A | 1.00 | 0.53 |

Note: Strength indicates the frequency with which an edge appeared in the bootstrap Bayesian networks. Direction indicates the frequency with which the edge in the Bayesian networks pointed in a given direction. Edges with strength >0.5 and direction >0.5 are shown in the table. In the final DAG, only edges with a strength greater than 85% are included. SPQ.CP = cognitive perceptual dimension of Schizotypal Personality Questionnaire; SPQ.I = interpersonal dimension of Schizotypal Personality Questionnaire; SPQ.D = disorganized dimension of Schizotypal Personality Questionnaire; ERQ.R= reappraisal dimension of Emotion Regulation Questionnaire; ERQ.S= suppression dimension of Emotion Regulation Questionnaire; DASS.D= depression dimension of Depression Anxiety Stress Scales; DASS.A= anxiety dimension of Depression Anxiety Stress Scales; DASS.S= stress dimension of Depression Anxiety Stress Scales;

**Table S1.8 Arc strength estimated from the Bayesian networks of the high schizotypy group**

| From | To | Strength | Direction |
| --- | --- | --- | --- |
| ERQ.R | ERQ.S | 1.00 | 0.93 |
| SPQ.I | ERQ.S | 0.92 | 0.89 |
| SPQ.I | DASS.D | 0.99 | 0.53 |
| DASS.D | ERQ.S | 0.85 | 0.95 |
| DASS.D | SPQ.D | 0.99 | 0.58 |
| DASS.D | DASS.A | 1.00 | 0.57 |
| DASS.D | DASS.S | 1.00 | 0.61 |
| DASS.A | SPQ.CP | 0.61 | 0.74 |
| DASS.S | SPQ.D | 0.86 | 0.58 |
| DASS.S | DASS.A | 1.00 | 0.52 |

Note: Strength indicates the frequency with which an edge appeared in the bootstrap Bayesian networks. Direction indicates the frequency with which the edge in the Bayesian networks pointed in a given direction. Edges with strength >0.5 and direction >0.5 are shown in the table. In the final DAG, only edges with a strength greater than 85% are included. SPQ.CP = cognitive perceptual dimension of Schizotypal Personality Questionnaire; SPQ.I = interpersonal dimension of Schizotypal Personality Questionnaire; SPQ.D = disorganized dimension of Schizotypal Personality Questionnaire; ERQ.R= reappraisal dimension of Emotion Regulation Questionnaire; ERQ.S= suppression dimension of Emotion Regulation Questionnaire; DASS.D= depression dimension of Depression Anxiety Stress Scales; DASS.A= anxiety dimension of Depression Anxiety Stress Scales; DASS.S= stress dimension of Depression Anxiety Stress Scales;

**Table S1.9 Arc strength estimated from the Bayesian networks of the low schizotypy group.**

| From | To | Strength | Direction |
| --- | --- | --- | --- |
| ERQ.R | ERQ.S | 1.00 | 0.95 |
| ERQ.S | SPQ.D | 0.56 | 0.73 |
| SPQ.I | ERQ.S | 0.66 | 0.91 |
| DASS.D | ERQ.S | 0.77 | 0.96 |
| DASS.D | SPQ.I | 0.73 | 0.60 |
| DASS.D | DASS.A | 1.00 | 0.60 |
| DASS.D | DASS.S | 0.99 | 0.63 |
| DASS.A | SPQ.CP | 0.82 | 0.60 |
| DASS.A | DASS.S | 1.00 | 0.57 |
| DASS.S | SPQ.CP | 0.53 | 0.53 |

Note: Strength indicates the frequency with which an edge appeared in the bootstrap Bayesian networks. Direction indicates the frequency with which the edge in the Bayesian networks pointed in a given direction. Edges with strength >0.5 and direction >0.5 are shown in the table. In the final DAG, only edges with a strength greater than 85% are included. SPQ.CP = cognitive perceptual dimension of Schizotypal Personality Questionnaire; SPQ.I = interpersonal dimension of Schizotypal Personality Questionnaire; SPQ.D = disorganized dimension of Schizotypal Personality Questionnaire; ERQ.R= reappraisal dimension of Emotion Regulation Questionnaire; ERQ.S= suppression dimension of Emotion Regulation Questionnaire; DASS.D= depression dimension of Depression Anxiety Stress Scales; DASS.A= anxiety dimension of Depression Anxiety Stress Scales; DASS.S= stress dimension of Depression Anxiety Stress Scales;

**Table S1.10 Arc strength estimated from the Bayesian networks of the 9-10 age group**

| From | To | Strength | Direction |
| --- | --- | --- | --- |
| ERQ.R | ERQ.S | 1.00 | 0.80 |
| SPQ.CP | SPQ-I | 1.00 | 0.68 |
| SPQ.CP | SPQ-D | 1.00 | 0.57 |
| SPQ.I | ERQ.S | 0.78 | 0.76 |
| DASS.D | ERQ.S | 0.72 | 0.96 |
| DASS.D | SPQ.I | 0.60 | 0.74 |
| DASS.D | SPQ.D | 0.91 | 0.70 |
| DASS.D | DASS.A | 1.00 | 0.56 |
| DASS.D | DASS.S | 1.00 | 0.50 |
| DASS.A | SPQ.CP | 0.67 | 0.60 |
| DASS.S | SPQ.CP | 0.92 | 0.65 |
| DASS.S | SPQ.I | 0.79 | 0.73 |
| DASS.S | SPQ.D | 0.90 | 0.70 |
| DASS.S | DASS.A | 1.00 | 0.59 |

Note: Strength indicates the frequency with which an edge appeared in the bootstrap Bayesian networks. Direction indicates the frequency with which the edge in the Bayesian networks pointed in a given direction. Edges with strength >0.5 and direction >0.5 are shown in the table. In the final DAG, only edges with a strength greater than 85% are included. SPQ.CP = cognitive perceptual dimension of Schizotypal Personality Questionnaire; SPQ.I = interpersonal dimension of Schizotypal Personality Questionnaire; SPQ.D = disorganized dimension of Schizotypal Personality Questionnaire; ERQ.R= reappraisal dimension of Emotion Regulation Questionnaire; ERQ.S= suppression dimension of Emotion Regulation Questionnaire; DASS.D= depression dimension of Depression Anxiety Stress Scales; DASS.A= anxiety dimension of Depression Anxiety Stress Scales; DASS.S= stress dimension of Depression Anxiety Stress Scales;

**Table S1.11 Arc strength estimated from the Bayesian networks of the 11-12 age group**

| From | To | Strength | Direction |
| --- | --- | --- | --- |
| ERQ.R | ERQ.S | 1.00 | 0.97 |
| SPQ.CP | SPQ-I | 1.00 | 0.83 |
| SPQ.CP | SPQ-D | 0.97 | 0.76 |
| SPQ.I | ERQ.S | 0.94 | 0.94 |
| DASS.D | ERQ.S | 0.70 | 0.98 |
| DASS.D | SPQ.I | 1.00 | 0.86 |
| DASS.D | SPQ.D | 0.92 | 0.80 |
| DASS.D | DASS.A | 1.00 | 0.53 |
| DASS.D | DASS.S | 1.00 | 0.67 |
| DASS.A | SPQ.CP | 1.00 | 0.56 |
| DASS.A | DASS.S | 1.00 | 0.66 |
| DASS.S | SPQ.I | 0.89 | 0.52 |
| DASS.S | SPQ.D | 0.72 | 0.75 |

Note: Strength indicates the frequency with which an edge appeared in the bootstrap Bayesian networks. Direction indicates the frequency with which the edge in the Bayesian networks pointed in a given direction. Edges with strength >0.5 and direction >0.5 are shown in the table. In the final DAG, only edges with a strength greater than 85% are included. SPQ.CP = cognitive perceptual dimension of Schizotypal Personality Questionnaire; SPQ.I = interpersonal dimension of Schizotypal Personality Questionnaire; SPQ.D = disorganized dimension of Schizotypal Personality Questionnaire; ERQ.R= reappraisal dimension of Emotion Regulation Questionnaire; ERQ.S= suppression dimension of Emotion Regulation Questionnaire; DASS.D= depression dimension of Depression Anxiety Stress Scales; DASS.A= anxiety dimension of Depression Anxiety Stress Scales; DASS.S= stress dimension of Depression Anxiety Stress Scales;
