# Supplementary material for: Relationship Between Schizotypal Traits, Emotion Regulation, and Negative Affect in Children: A Network Analysis
Source: Schizophr Bull. 2025 Mar 4;51(Suppl 2):S226–37. doi: 10.1093/schbul/sbae172 (PMC11879503; doi:10.1093/schbul/sbae172)
Supplement: sbae172_suppl_Supplementary_Materials [file sbae172_suppl_supplementary_materials.zip › Supplementary Materials_2 13_08_24.docx]

**Supplementary Materials_2**

**Relationship between schizotypal traits, emotion regulation and negative affect in children: A network analysis**

**Sensitivity analysis**

**Supplementary Materials_2**

**S2.1. Results**

**S2.2.** **Sensitivity Analysis Results for the Whole Sample**

**S2.2.1** Sensitivity Analysis for Whole-Sample Network Analysis

**Table S2.1** Descriptive characteristics of the whole sample without outliers

**Figure S2.1** Regularized partial correlation network and the standardized centrality estimates for the whole sample

**Figure S2.2** Bootstrapped 95% confidence intervals of edge weights and the stability of Centrality indicators of the whole-sample network without outliers

**Figure S2.3** Bootstrapped difference tests between edge-weights in the network of whole sample

**Figure S2.4** Bootstrapped difference test for node centrality indices in the network of whole sample without outliers

**S2.2.2** Sensitivity Analysis Results Results for Bridge Centrality

**Figure S2.5** The standardized bridge centrality estimates of the whole-sample network

**Figure S2.6** Bootstrapped difference test for bridge centrality indices in the network of whole sample without outliers

**Figure S2.7** The stability of bridge centrality indicators computed by case-dropping bootstrap

**S2.3 Sensitivity Analysis Results for Gender, Age, and High/Low Group Differences**

**S2.3.1** Figures for Network Comparisons

**Figure S2.8** Regularized partial networks in boys and girls

**Figure S2.9** Regularized partial networks in children aged 9-10 years and 11-12 years

**Figure S2.10** Regularized partial networks in low schizotypy and high schizotypy groups

**S2.3.2** Tables for Independent sample t tests

**Table S2.2** Gender difference in age, schizotypal traits, emotion regulation and negative affect

**Table S2.3** Age-group difference in schizotypal traits, emotion regulation and negative affect

**Table S2.4** High/Low schizotypy difference in age, schizotypal traits, emotion regulation and negative affect

**S2.4 Sensitivity Analysis Results for Bayesian network**

**S2.4.1 Figures for** **Bayesian networks**

**Figure S2.11** A Bayesian network for the whole sample (directed acyclic graph; DAG)

**Figure S2.12** Estimated Bayesian networks for the low and high schizotypy groups

**Figure S2.13** Estimated Bayesian networks in children aged 9-10 years and 11-12 years

**S1.3.2 Tables for Bayesian networks**

**Table S2.5** Arc strength estimated from the Bayesian networks of the whole sample **Table S2.6** Arc strength estimated from the Bayesian networks of the high schizotypy group

**Table S2.7** Arc strength estimated from the Bayesian networks of the low schizotypy group

**Table S2.8** Arc strength estimated from the Bayesian networks of the 9-10 age group

**Table S2.9** Arc strength estimated from the Bayesian networks of the 11-12 age group

**S2.1. Results**

In this section, we attempted to identify potential outliers using the interquartile range (IQR) method as the safeguard standard technique in the present study. This is a commonly used approach, and it is suggests that the IQR method exhibits good hit rates when sample sizes are larger (n≥32) and the proportion of outliers is low (<25%) (Jones, 2019). We examined the interquartile range (IQR), i.e., the range between the first quartile (Q1) and the third quartile (Q3), of subscale scores, and found 112 subjects who scored beyond the lower (the Q1 - 1.5*IQR) or the upper (Q3 + 1.5*IQR) arbitrary “outliers’ cut-off” values in any of the subscale scores, and excluded them as outliers.

In this section, we first present the main results without outliers, corresponding to the main text, including network analysis, network comparison, bridge analysis, and the results of Directed Acyclic Graphs (DAG). Next, we compared the networks with and without outliers.

**S2.1.1 The LASSO network**

As shown in Figure S2.1(A), we observed significant positive correlations between three dimension of schizotypal traits (i.e., the cognitive-perceptual, interpersonal, and disorganized subscales) and negative affect (i.e., depressed mood, anxiety, and stress) (*edge values*= 0.03-0.20). Moreover, the interpersonal and the disorganized dimensions of schizotypal traits were positively correlated with emotion suppression (*edge values* = 0.08 and 0.03, respectively). The cognitive-perceptual dimension was positively correlated with emotion reappraisal (*edge value* = 0.02). Positive correlations were found between emotion suppression and negative affect (i.e., depressed mood, anxiety, and stress) (*edge values* = 0.01-0.11). Negative correlations were found between emotion reappraisal and the negative affect of depressed mood and stress (*edge values* = -0.10, and -0.04 respectively). According to the difference test for edge weights in Figure S2.3, the correlation between SPQ-CP and anxiety is significantly greater than that between SPQ-D, SPQ-I, and anxiety.

**S2.1.2 Network inference**

Centrality indices are shown in FigureS2.1(B). Based on the difference test for node centrality in Figure S2.4, stress and depressed mood has significantly higher strength than all nodes except anxiety. Stress has significantly higher EI than everty other nodes. Stress displayed higher closeness centrality in comparison to emotion suppression, emotion reappraisal, and SPQ-D, but not significantly higher than the remaining nodes. However, the stability of betweenness was low. The predictability of nodes ranged from 27% (emotion reappraisal) to 60% (stress), with an average of 43%.

**S2.1.3 Bridging nodes**

As shown in Figure S2.5, in the bridge centrality network, depressed mood and stress (nodes of negative affect) showed the highest bridge strength, with 80th quantile cut-off. On the other hand, stress and the cognitive-perceptual dimension of schizotypal traits showed the highest bridge EI (1-level), with 80th quantile cut-off. Stress and the interpersonal dimension of schizotypal traits showed the highest bridge closeness, with 80th quantile cut-off. However, according to the difference test for bridge node centrality in Figure S2.6, most differences were not statistically significant. The notable exception was the significantly lower bridge EI of emotion reappraisal compared to every other node, highlighting its distinct role.

**S2.1.4 NCT results between boys and girls**

As shown in Table S2.2, independent t-tests revealed that girls scored higher than boys in the interpersonal dimension of schizotypal traits, as well as in emotion suppression and reappraisal, and negative affect (depressed mood, anxiety, and stress). **However, no significant gender difference was found in the cognitive-perceptual and the disorganized dimensions of schizotypal traits.**

Figure S2.8(A) and Figure S2.8(B) showed estimated gender-stratified networks. NCT found the two gender-stratified networks having comparable network structure (i.e., the maximum difference in edge-weights was 0.15, p = 0.30) and global strength (i.e., the strength difference was 0.19, p = 0.41, global strength for boys’ network is 3.37 and 3.56 for girls’ network). We did not find any significant gender difference when comparing the edge-weights and (all *ps* > 0.05) and centrality of nodes (all *ps* > 0.05).

**S2.1.5 NCT results between boys and girls**

For age effect, independent sample t tests (see Table S2.3) showed that children aged 11-12 showed no significant differences compared to children aged 9-10. The regularized partial correlation networks for children aged 9-10 and 11-12 are shown in S2.9 (A) and S2.9 (B). NCT showed significant differences in global strength (i.e., the strength difference was 0.25, p = 0.26, with the global strength being 3.17 for the 9-10 network and 3.42 for the 11-12 network), and the network structure (i.e., the maximum difference in edge-weights was 0.22, *p* = 0.01). Additionally, NCT found children aged 11-12 have a stronger correlation between SPQ-CP and anxiety compared to children aged 9-10 (*p* = 0.02).

**S2.1.6 NCT results between high and low schizotypy groups**

As shown in Table S2.4, the high schizotypy group exhibited not only higher levels of schizotypal traits but also higher levels of negative affect, as well as increased use of both suppression and reappraisal compared to the low schizotypy group. Figures S2.10 (A) and S2.10 (B) display regularized partial correlation networks for two groups). NCT showed significant differences in the network structure (i.e., the maximum difference in edge-weights was 0.28, p < 0.001), but not in global strength (i.e., the strength difference was 0.24, p = 0.65, with the global strength being 2 for the high schizotypy group and 1.76 for the low schizotypy group)

In addition, the low schizotypy group demonstrated higher strength in SPQ-CP, suppression and reappraisal, as well as higher EI of SPQ-CP and reappraisal than the high schizotypy group. NCT also found significant differences in edge weights after FDR correction, including Reappraisal- Suppression (p < 0.01).

**S2.1.7 Network stability and accuracy**

As shown in Figures S2.2(A) and S2.2(B), the bootstrapped confidence intervals (CI) of edge-weights were narrow. The correlation stability (CS)-coefficients for strength, closeness and expected influence (EI) were 0.75, 0.67 and 0.75 respectively, indicating that these centrality indices were stable. The CS-coefficients for bridge strength and bridge expected influence (EI) were 0.52 and 0.75 respectively, indicating these bridge centrality indices were stable (see Figure S2.7 in the Supplementary Materials). However, the CS-coefficients for betweenness, bridge betweenness and bridge closeness are below the recommended threshold (> 0.5).

**S2.1.8 The DAG network**

Figure S2.11 showed the DAG network including schizotypal traits, negative affect and emotion regulation. In the DAG network, nodes located upstream (near the top) could be regarded as sources of activation to drive other nodes. As shown in Figure S2.11, stress was pointing to three dimensions of schizotypal traits. Anxiety had an edge pointing towards the SPQ-CP, and depressed mood had an edge pointing towards the SPQ-D. Furthermore, the SPQ-I had two edges pointing towards emotion suppression and depressed mood.

Given the significant differences found in the network comparisons between age groups and high/low schizotypy groups, we also conducted DAGs for these groups separately. In the DAG for the high schizotypy group, we observed depressed mood was pointing to SPQ-D. Additionally, SPQ-I had an edge pointing towards depression with a DAG edge strength of 83% (very close to the 85% threshold). In the DAG for the low-score group, we found that depressed mood appeared to drive SPQ-I, anxiety and stress. Anxiety was pointing to both SPQ-CP and stress. (see Figure S2.12). In the DAG for children aged 9-10, stress was pointing towards both SPQ-D and SPQ-CP. For children aged 11-12, the DAG showed that SPQ-I pointing to depressed mood, anxiety pointed to SPQ-CP, and stress pointed to SPQ-I (see Figure S2.13).

**S2.1.9 Network differences in networks with and without outliers**

We did not find any significant difference between the network structure (*M* = 0.05, *p* = 1), global strength (*S* = 0.17, *p* = 0.34), edge-weights (all *ps* > 0.05) and the centrality of nodes (all *ps* > 0.05).

**S2.2. Sensitivity Analysis Results for the Whole Sample**

**S1.2.1 Sensitivity Analysis for Whole-Sample Network Analysis**

**Table S2.1 Descriptive characteristics of the whole sample without outliers**

| Variables and the dimensions  (N=907) | Minimum | Maximum | Mean | Standard deviation | Skewness | Kurtosis |
| --- | --- | --- | --- | --- | --- | --- |
| Age (years) | 9 | 12 | 10.39 | 0.95 | 0.07 | -0.93 |
| Length of education (years) | 4 | 6 | 5 | 0.81 | -0.004 | -1.49 |
| SPQ-C total score  SPQ-C cognitive perceptual  SPQ-C interpersonal  SPQ-C disorganized  ERQ-CA total score  ERQ-CA reappraisal  ERQ-CA suppression  DASS-21 total score  DASS-21 depression  DASS-21 anxiety  DASS-21 stress | 0 | 20 | 5.55 | 4.13 | 0.49 | -0.52 |
|  | 0 | 8 | 2.60 | 1.99 | 0.46 | -0.60 |
|  | 0 | 8 | 2.10 | 1.89 | 0.64 | -0.40 |
|  | 0 | 5 | 0.85 | 1.17 | 1.51 | 1.80 |
|  |  |  |  |  |  |  |
|  | 10 | 50 | 28.19 | 9.65 | -0.12 | -0.56 |
|  | 6 | 30 | 18.23 | 6.81 | -0.16 | -0.93 |
|  | 4 | 20 | 9.96 | 4.26 | 0.39 | -0.61 |
|  |  |  |  |  |  |  |
|  | 0 | 32 | 8.18 | 7.24 | 0.92 | 0.15 |
|  | 0 | 10 | 2.00 | 2.45 | 1.39 | 1.37 |
|  | 0 | 11 | 3.17 | 2.91 | 0.89 | -0.02 |
|  | 0 | 12 | 3.01 | 2.92 | 0.88 | -0.04 |

Note: SPQ-C: Schizotypal Personality Questionnaire- Children; ERQ-CA: Emotion Regulation Questionnaire for Children and Adolescent; DASS-21: The Depression Anxiety Stress Scale

| (A) 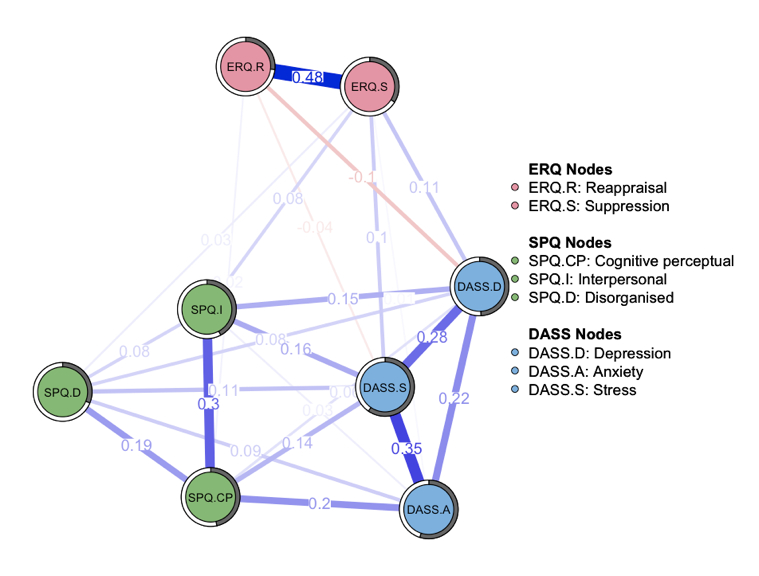 | (B)  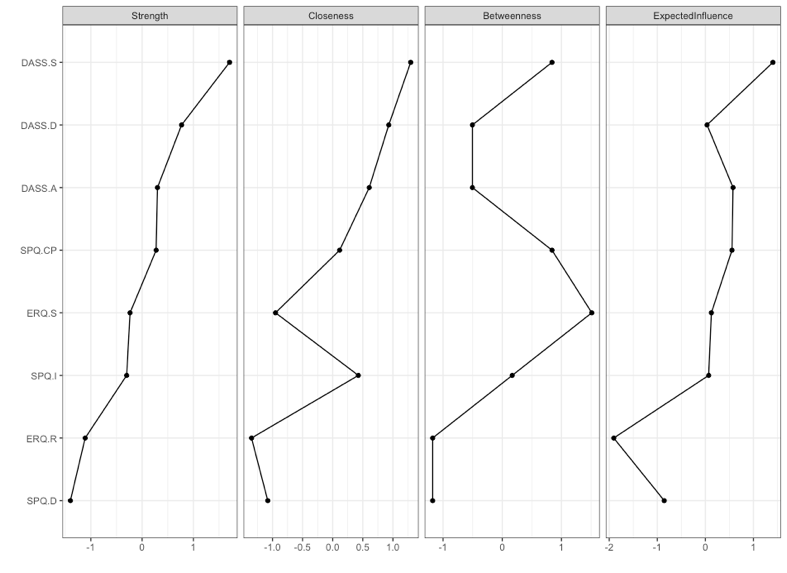 |
| --- | --- |
| **Figure S2.1** (A) Regularized partial correlation network for the whole sample without outliers. Each node represents a variable. Each edge represents the partial correlation between two nodes controlled for all other nodes. Thicker lines represent stronger connections. The value of each edge represents the strength of the partial correlations. The blue lines indicate positive partial correlations and the red lines indicate negative partial correlations. The blue ring around each node represents the predictability values, which indicates prediction of a specific node by other nodes in the network. (B)The standardized centrality estimates of the whole-sample network without outliers.  Note: SPQ.CP = cognitive perceptual dimension of Schizotypal Personality Questionnaire; SPQ.I = interpersonal dimension of Schizotypal Personality Questionnaire; SPQ.D = disorganized dimension of Schizotypal Personality Questionnaire; ERQ.R= reappraisal dimension of Emotion Regulation Questionnaire; ERQ.S= suppression dimension of Emotion Regulation Questionnaire; DASS.D= depression dimension of Depression Anxiety Stress Scales; DASS.A= anxiety dimension of Depression Anxiety Stress Scales; DASS.S= stress dimension of Depression Anxiety Stress Scales; | |

| (A)  **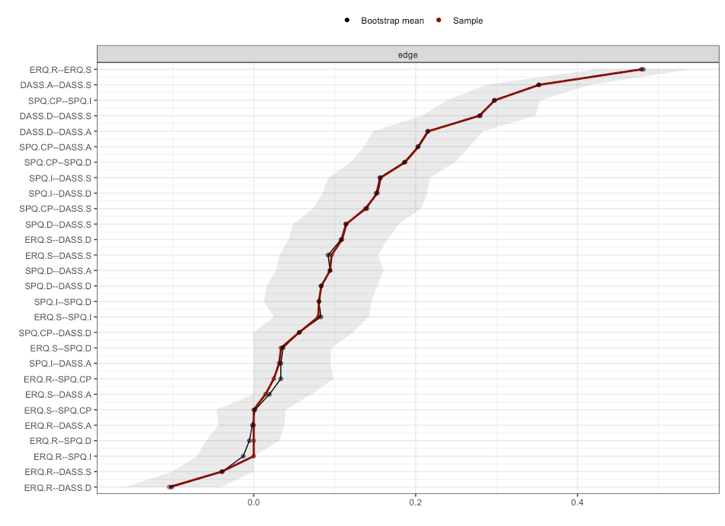** | (B)  **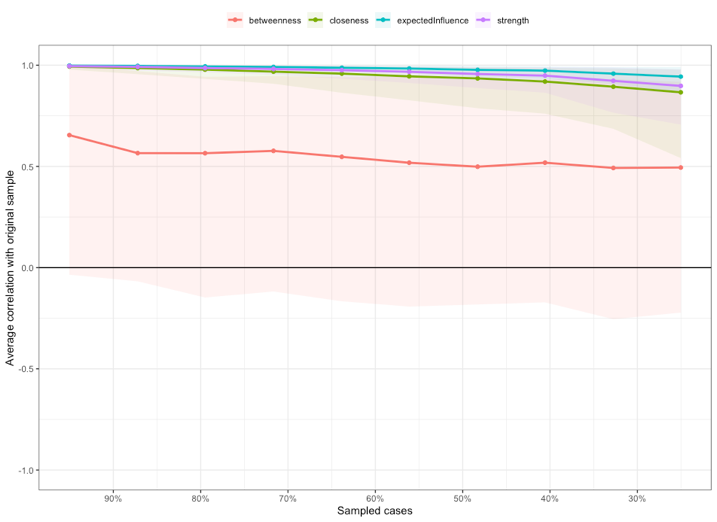** |
| --- | --- |
| **Figure S2.2** (A) Bootstrapped 95% confidence intervals of edge weights of the whole-sample network without outliers. The grey bar represents bootstrapped confidence interval and the red line represents the edge-weight. (B) The stability of Centrality indicators computed by case-dropping bootstrap. The lines represent the average correlation between the central indicators estimated from the bootstrapping subsamples and the central indicators estimated from the original sample. The areas represent a range from 2.5th quantiles to 97.5th quantiles. | |

| **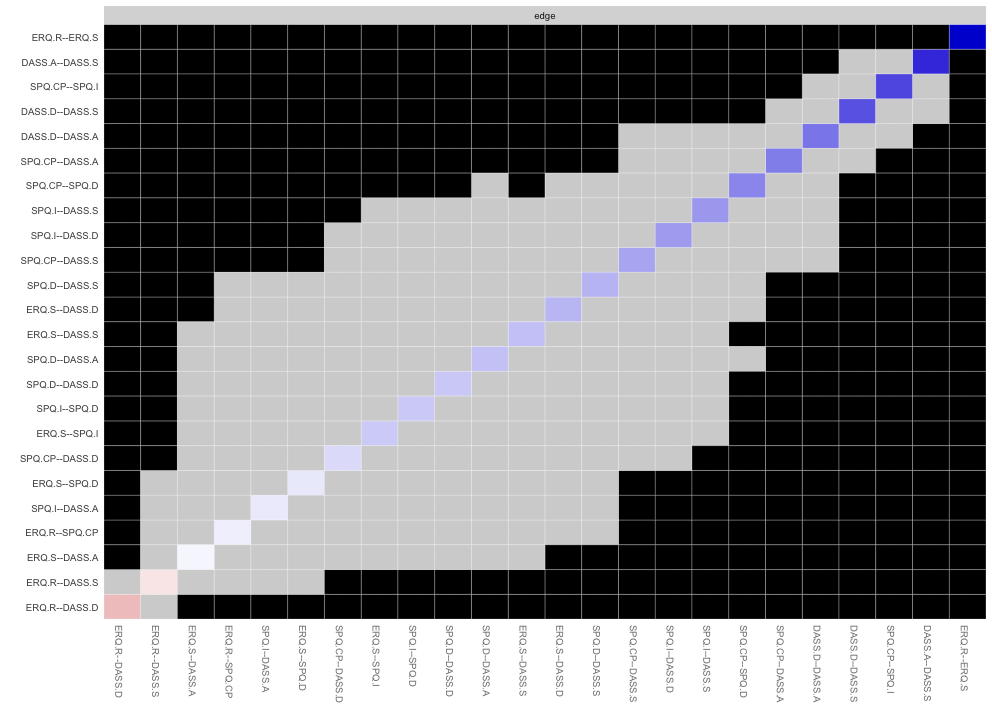** |
| --- |
| **Figure S2.3 Bootstrapped difference tests between edge-weights in the network of whole sample**  Note: Black boxes show edges that differed significantly form one another edges, and grey boxes show edges that were not significantly different. SPQ.CP = cognitive perceptual dimension of Schizotypal Personality Questionnaire; SPQ.I = interpersonal dimension of Schizotypal Personality Questionnaire; SPQ.D = disorganized dimension of Schizotypal Personality Questionnaire; ERQ.R= reappraisal dimension of Emotion Regulation Questionnaire; ERQ.S= suppression dimension of Emotion Regulation Questionnaire; DASS.D= depression dimension of Depression Anxiety Stress Scales; DASS.A= anxiety dimension of Depression Anxiety Stress Scales; DASS.S= stress dimension of Depression Anxiety Stress Scales; |

| **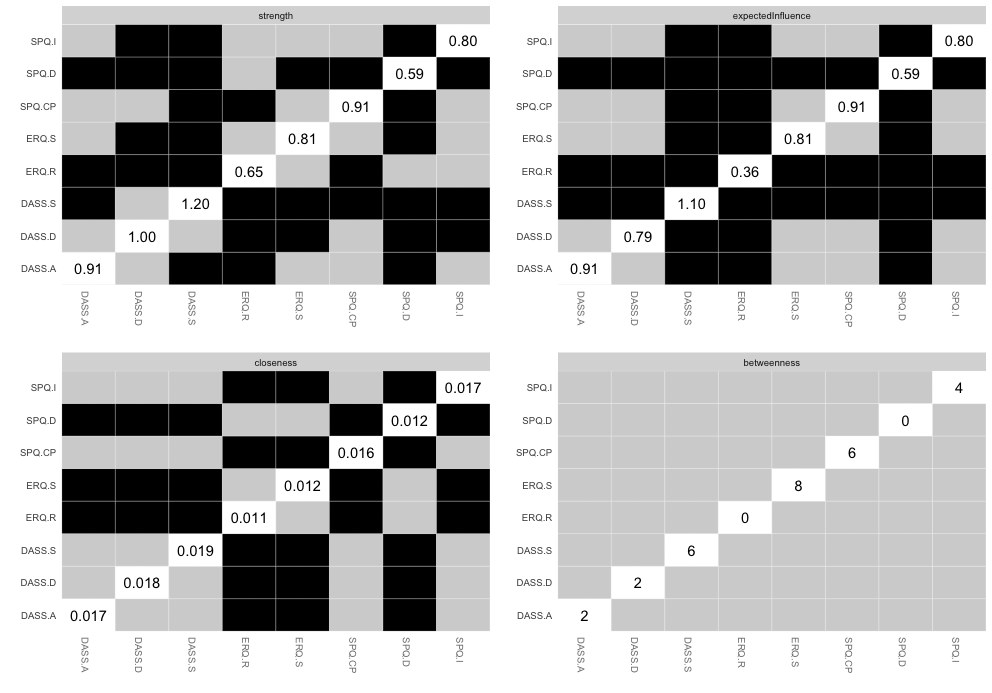** |
| --- |
| **Figure S2.4 Bootstrapped difference test for node centrality indices (from left to right and top to bottom: strength, EI, closeness, betweenness) in the network of whole sample without outliers**  Note: Black boxes show strengths of nodes that differed significantly form one another, while grey boxes shows strength of nodes that were not significantly different. SPQ.CP = cognitive perceptual dimension of Schizotypal Personality Questionnaire; SPQ.I = interpersonal dimension of Schizotypal Personality Questionnaire; SPQ.D = disorganized dimension of Schizotypal Personality Questionnaire; ERQ.R= reappraisal dimension of Emotion Regulation Questionnaire; ERQ.S= suppression dimension of Emotion Regulation Questionnaire; DASS.D= depression dimension of Depression Anxiety Stress Scales; DASS.A= anxiety dimension of Depression Anxiety Stress Scales; DASS.S= stress dimension of Depression Anxiety Stress Scales; |

**S2.2.2 Sensitivity Analysis Results Results for Bridge Centrality**

| 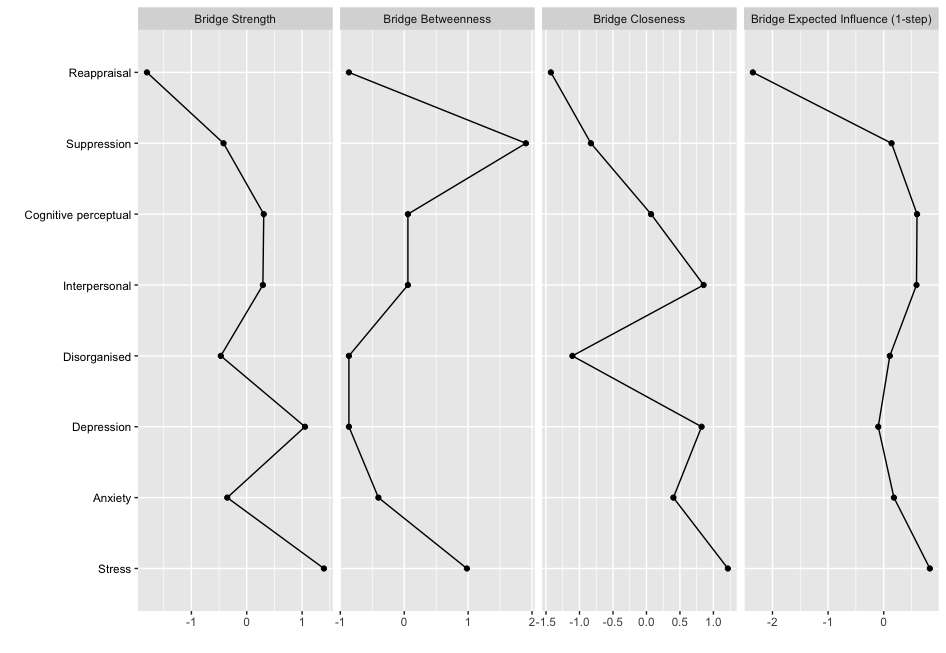 |
| --- |
| **Figure S2.5 The standardized bridge centrality estimates of the whole-sample network.** |

| 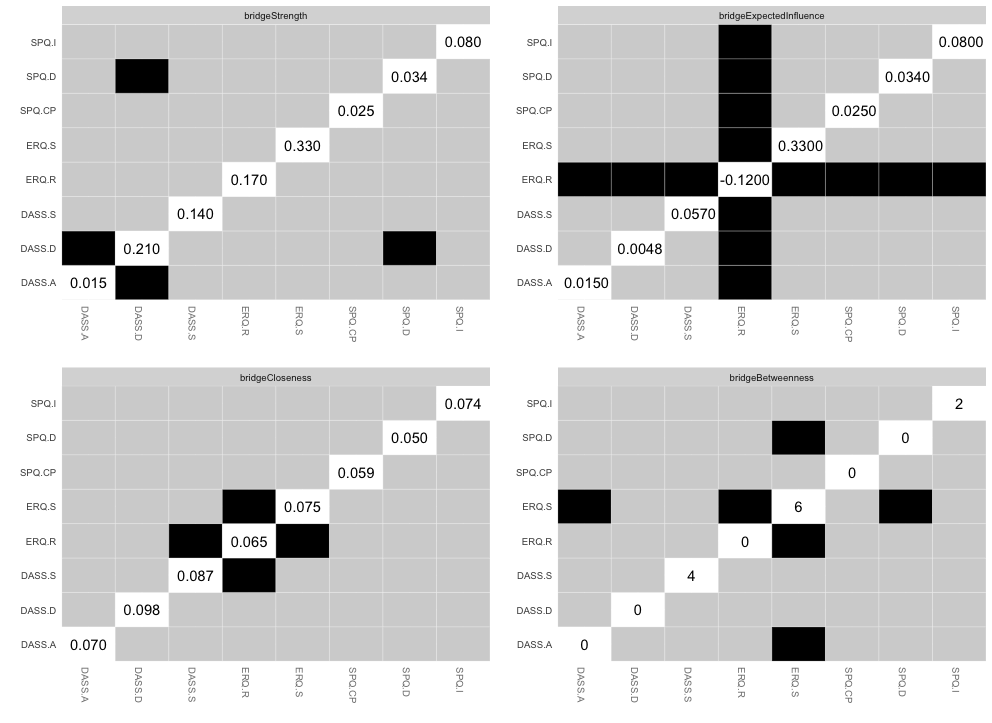 |
| --- |
| **Figure S2.6 Bootstrapped difference test for bridge centrality indices (from left to right and top to bottom: bridgeStrength, bridgeEI, bridgeCloseness, bridgeBetweenness) in the network of whole sample without outliers**  Note: Black boxes shows strengths of nodes that differed significantly form one another, while grey boxes shows strength of nodes that were not significantly different. SPQ.CP = cognitive perceptual dimension of Schizotypal Personality Questionnaire; SPQ.I = interpersonal dimension of Schizotypal Personality Questionnaire; SPQ.D = disorganized dimension of Schizotypal Personality Questionnaire; ERQ.R= reappraisal dimension of Emotion Regulation Questionnaire; ERQ.S= suppression dimension of Emotion Regulation Questionnaire; DASS.D= depression dimension of Depression Anxiety Stress Scales; DASS.A= anxiety dimension of Depression Anxiety Stress Scales; DASS.S= stress dimension of Depression Anxiety Stress Scales; |

| 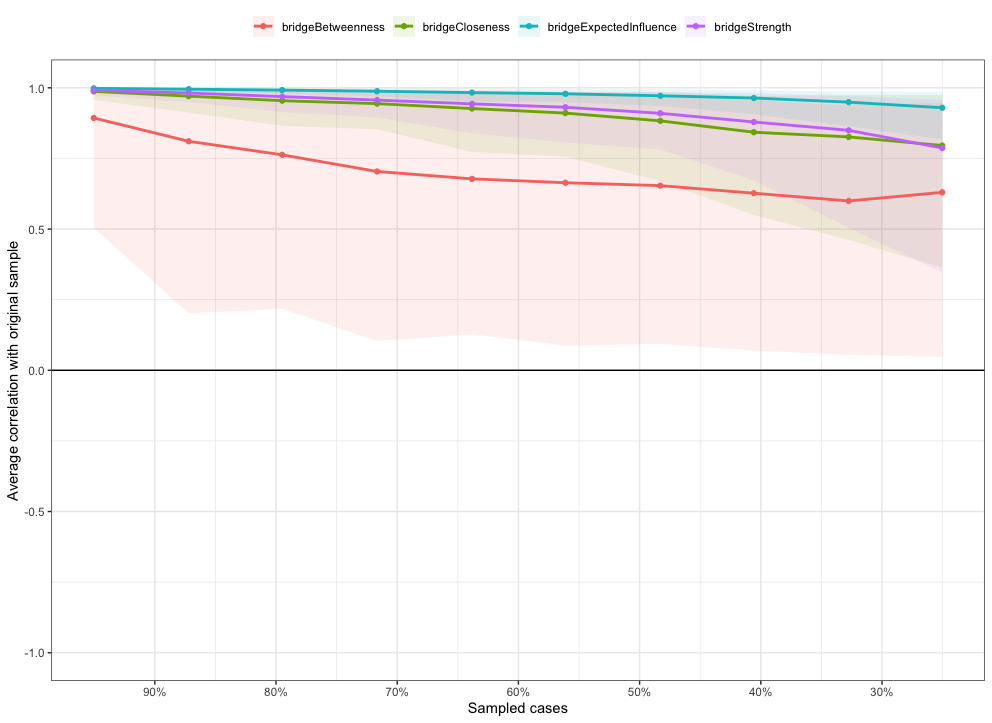 |
| --- |
| **Figure S2.7 The stability of bridge centrality indicators computed by case-dropping bootstrap.**  Note: The lines represent the average correlation between the central indicators estimated from the bootstrapping subsamples and the bridge central indicators estimated from the original sample. The areas represent a range from 2.5th quantiles to 97.5th quantiles. |

**S2.3 Sensitivity Analysis Results for Gender, Age, and High/Low Group Differences**

**S1.3.1 Figures for Network Comparisons**

| 1. Boys   **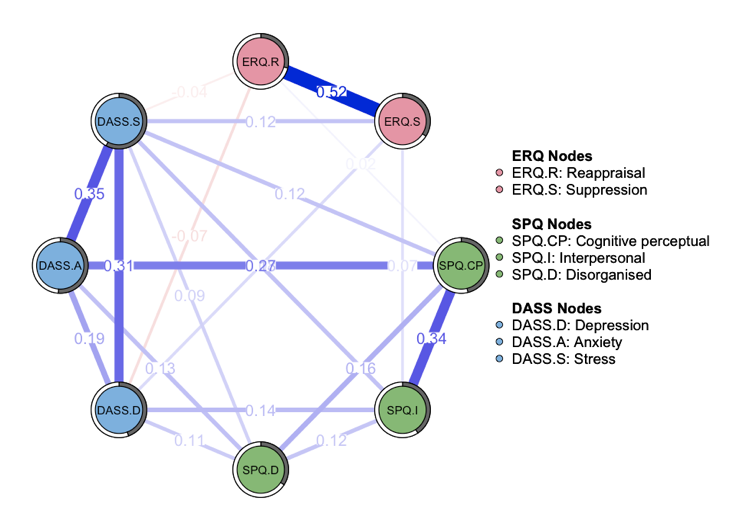** | 1. Girls   **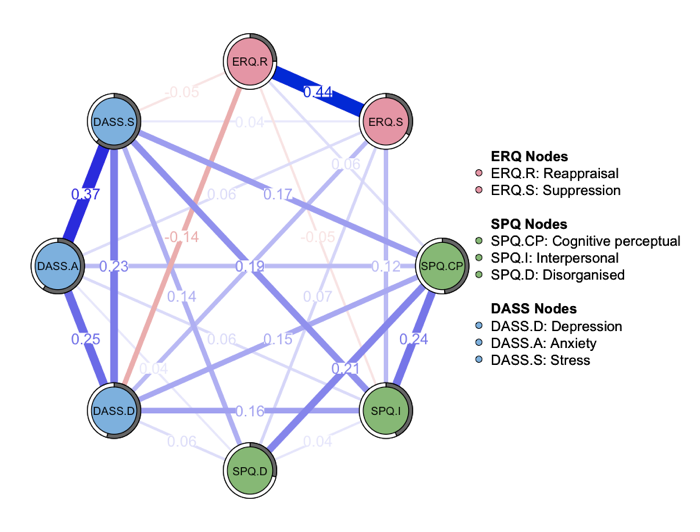** |
| --- | --- |
| **Figure S2.8 Regularized partial networks in boys (left, n=493) and girls (right, n=414).**  Each node represents a variable. Each edge represents the partial correlation between two nodes controlled for all other nodes. Thicker lines represent stronger connections. The value of each edge represents the strength of the partial correlations. The blue lines indicate positive partial correlations and the red lines indicate negative partial correlations. The blue ring around each node represents the predictability values, which indicates prediction of a specific node by other nodes in the network.  Note: SPQ.CP = cognitive perceptual dimension of Schizotypal Personality Questionnaire; SPQ.I = interpersonal dimension of Schizotypal Personality Questionnaire; SPQ.D = disorganized dimension of Schizotypal Personality Questionnaire; ERQ.R= reappraisal dimension of Emotion Regulation Questionnaire; ERQ.S= suppression dimension of Emotion Regulation Questionnaire; DASS.D= depression dimension of Depression Anxiety Stress Scales; DASS.A= anxiety dimension of Depression Anxiety Stress Scales; DASS.S= stress dimension of Depression Anxiety Stress Scales; | |

| (A) 9-11 age group  **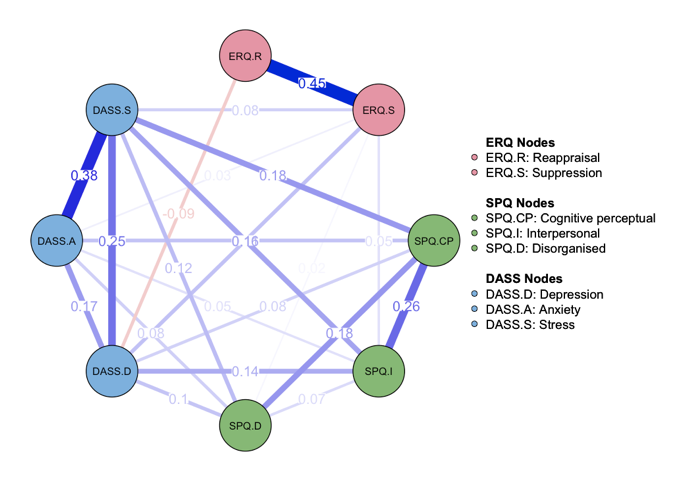** | (B) 11-12 age group  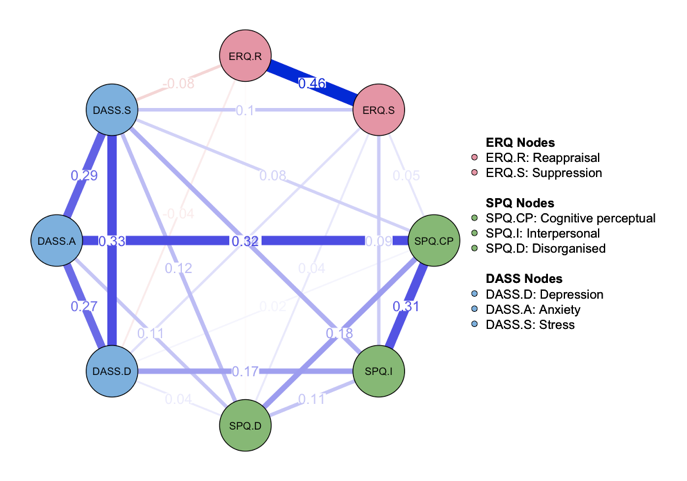 |
| --- | --- |
| **Figure S2.9 Regularized partial networks in children aged 9-10 years (left, n=490) and 11-12 years (right, n=417).** Each node represents a variable. Each edge represents the partial correlation between two nodes controlled for all other nodes. Thicker lines represent stronger connections. The value of each edge represents the strength of the partial correlations. The blue lines indicate positive partial correlations and the red lines indicate negative partial correlations. The blue ring around each node represents the predictability values, which indicates prediction of a specific node by other nodes in the network.  Note: SPQ.CP = cognitive perceptual dimension of Schizotypal Personality Questionnaire; SPQ.I = interpersonal dimension of Schizotypal Personality Questionnaire; SPQ.D = disorganized dimension of Schizotypal Personality Questionnaire; ERQ.R= reappraisal dimension of Emotion Regulation Questionnaire; ERQ.S= suppression dimension of Emotion Regulation Questionnaire; DASS.D= depression dimension of Depression Anxiety Stress Scales; DASS.A= anxiety dimension of Depression Anxiety Stress Scales; DASS.S= stress dimension of Depression Anxiety Stress Scales; | |

| (A) Low schizotypy  **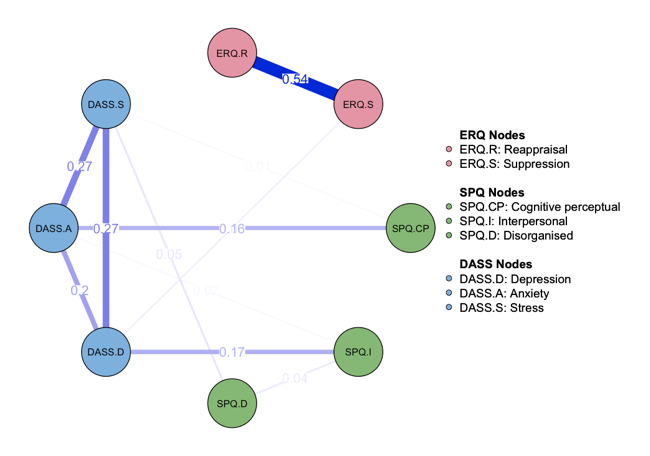** | (B) High schizotypy  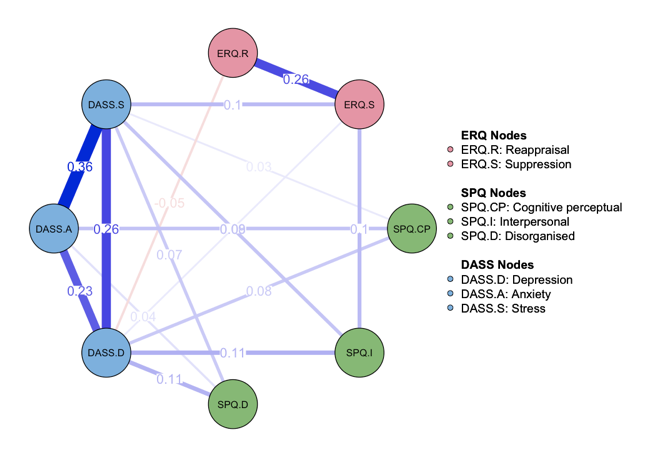 |
| --- | --- |
| **Figure S2.10 Regularized partial networks in low schizotypy (left, n=401) and high schizotypy (right, n=430) groups.**  Each node represents a variable. Each edge represents the partial correlation between two nodes controlled for all other nodes. Thicker lines represent stronger connections. The value of each edge represents the strength of the partial correlations. The blue lines indicate positive partial correlations and the red lines indicate negative partial correlations. The blue ring around each node represents the predictability values, which indicates prediction of a specific node by other nodes in the network.  Note: SPQ.CP = cognitive perceptual dimension of Schizotypal Personality Questionnaire; SPQ.I = interpersonal dimension of Schizotypal Personality Questionnaire; SPQ.D = disorganized dimension of Schizotypal Personality Questionnaire; ERQ.R= reappraisal dimension of Emotion Regulation Questionnaire; ERQ.S= suppression dimension of Emotion Regulation Questionnaire; DASS.D= depression dimension of Depression Anxiety Stress Scales; DASS.A= anxiety dimension of Depression Anxiety Stress Scales; DASS.S= stress dimension of Depression Anxiety Stress Scales; | |

**S2.3.2 Tables for Independent sample t tests**

**Table S2.2** *Gender difference in age, schizotypal traits, emotion regulation and negative affect*

|  | Sample  (Mean ± standard deviation) | | *t* | *df* | *p* | *Cohen's d* |
| --- | --- | --- | --- | --- | --- | --- |
|  | Boys  (n=493) | Girls  (n=414) |  |  |  |  |
| Age (years) | 10.36±0.97 | 10.43±0.93 | -1.15 | 905 | 0.25 | -0.08 |
| Length of education (years) | 4.95±0.83 | 5.07±0.80 | -2.22 | 905 | 0.03 | -0.15 |
|  |  |  |  |  |  |  |
| SPQ-C total score | 5.26±4.06 | 5.90±4.18 | -2.34 | 905 | 0.02 | -0.16 |
| SPQ-C cognitive perceptual | 2.50±2.00 | 2.71±1.97 | -1.60 | 905 | 0.11 | -0.11 |
| SPQ-C interpersonal | 1.96±1.77 | 2.28±2.02 | -2.51 | 883.50 | 0.01 | -0.17 |
| SPQ-C disorganized | 0.80±1.19 | 0.91±1.13 | -1.46 | 905 | 0.15 | -0.10 |
|  |  |  |  |  |  |  |
| ERQ-CA total score | 27.17±9.89 | 29.41±9.22 | -3.52 | 895.06 | <.001 | -0.23 |
| ERQ-CA suppression | 9.41±4.16 | 10.60±4.29 | -4.24 | 905 | <.001 | -0.28 |
| ERQ-CA cognitive reappraisal | 17.76±7.02 | 18.80±6.51 | -2.32 | 896.27 | 0.02 | -0.15 |
|  |  |  |  |  |  |  |
| DASS-21 total score | 7.37±6.81 | 9.15±7.61 | -3.67 | 837.41 | <.001 | -0.25 |
| DASS-21 depression | 1.73±2.22 | 2.31±2.67 | -3.50 | 805.80 | <.001 | -0.24 |
| DASS-21 anxiety | 2.89±2.88 | 3.51±2.93 | -3.19 | 905 | 0.001 | -0.21 |
| DASS-21 stress | 2.75±2.82 | 3.33±3.00 | -3.01 | 905 | 0.003 | -0.20 |

Note: SPQ-C: Schizotypal Personality Questionnaire- Children; ERQ-CA: Emotion Regulation. Questionnaire for Children and Adolescent; DASS-21: The Depression Anxiety Stress Scales.If Levene's test indicated that the variances were equal across the two groups (p>0.05), we reported the results of the t-test assuming equal variances. Conversely, if Levene's test indicated that the variances were not equal across the two groups (p<0.05), we reported the results of the t-test assuming unequal variances.

**Table S2.3** *Age-group difference in schizotypal traits, emotion regulation and negative affect*

|  | Sample  (Mean ± standard deviation) | | *t* | *df* | *p* | *Cohen's d* |
| --- | --- | --- | --- | --- | --- | --- |
|  | 9-10  (n=490) | 11-12  (n=417) |  |  |  |  |
| Age (years) | 9.63±0.48 | 11.28±0.45 | -53.29 | 897.65 | <.001 | -3.53 |
| Length of education (years) | 4.40±0.49 | 5.71±0.47 | -40.98 | 890.28 | <.001 | -2.72 |
|  |  |  |  |  |  |  |
| SPQ-C total score | 5.69±4.02 | 5.38±4.24 | 1.13 | 905 | 0.26 | 0.08 |
| SPQ-C cognitive perceptual | 2.68±1.98 | 2.50±2.00 | 1.41 | 905 | 0.16 | 0.09 |
| SPQ-C interpersonal | 2.19±1.87 | 2.01±1.92 | 1.44 | 905 | 0.15 | 0.10 |
| SPQ-C disorganized | 0.82±1.17 | 0.88±1.17 | -0.74 | 882.02 | 0.46 | -0.05 |
|  |  |  |  |  |  |  |
| ERQ-CA total score | 28.46±9.65 | 27.87±9.66 | 0.92 | 905 | 0.36 | 0.06 |
| ERQ-CA suppression | 10.03±4.27 | 9.87±4.25 | 0.56 | 905 | 0.58 | 0.04 |
| ERQ-CA cognitive reappraisal | 18.43±6.79 | 18.00±6.83 | 0.95 | 905 | 0.34 | 0.06 |
|  |  |  |  |  |  |  |
| DASS-21 total score | 8.16±7.00 | 8.21±7.51 | -0.10 | 905 | 0.92 | -0.01 |
| DASS-21 depression | 1.89±2.32 | 2.13±2.59 | -1.46 | 843.49 | 0.14 | -0.10 |
| DASS-21 anxiety | 3.20±2.92 | 3.14±2.91 | 0.31 | 905 | 0.75 | 0.02 |
| DASS-21 stress | 3.07±2.88 | 2.94±2.96 | 0.66 | 905 | 0.51 | 0.04 |

Note: SPQ-C: Schizotypal Personality Questionnaire- Children; ERQ-CA: Emotion Regulation. Questionnaire for Children and Adolescent; DASS-21: The Depression Anxiety Stress Scales.If Levene's test indicated that the variances were equal across the two groups (p>0.05), we reported the results of the t-test assuming equal variances. Conversely, if Levene's test indicated that the variances were not equal across the two groups (p<0.05), we reported the results of the t-test assuming unequal variances.

**Table S2.4** *High/Low schizotypy difference in age, schizotypal traits, emotion regulation and negative affect*

|  | Sample  (Mean ± standard deviation) | | *t* | *df* | *p* | *Cohen's d* |
| --- | --- | --- | --- | --- | --- | --- |
|  | Low  (n=401) | High  (n=430) |  |  |  |  |
| Age (years) | 10.44±0.91 | 10.36±0.99 | 1.23 | 828.92 | 0.22 | 0.09 |
| Length of education (years) | 5.05±0.80 | 4.99±0.82 | 1.09 | 829 | 0.28 | 0.08 |
|  |  |  |  |  |  |  |
| SPQ-C total score | 1.75±1.40 | 9.19±2.71 | -50.14 | 652.48 | <.001 | -3.41 |
| SPQ-C cognitive perceptual | 0.95±0.97 | 4.17±1.52 | -36.58 | 736.29 | <.001 | -2.50 |
| SPQ-C interpersonal | 0.62±0.88 | 3.50±1.62 | -32.10 | 672.68 | <.001 | -2.19 |
| SPQ-C disorganized | 0.18±0.43 | 1.52±1.31 | -20.18 | 524.24 | <.001 | -1.36 |
|  |  |  |  |  |  |  |
| ERQ-CA total score | 26.31±10.66 | 29.73±8.27 | -5.15 | 753.45 | <.001 | -0.36 |
| ERQ-CA suppression | 8.69±4.21 | 11.01±3.97 | -8.19 | 829 | <.001 | -0.57 |
| ERQ-CA cognitive reappraisal | 17.62±7.52 | 18.72±6.03 | -2.32 | 766.96 | 0.02 | -0.16 |
|  |  |  |  |  |  |  |
| DASS-21 total score | 3.28±3.64 | 12.86±7.03 | -24.90 | 653.33 | <.001 | -1.69 |
| DASS-21 depression | 0.70±1.20 | 3.24±2.74 | -17.46 | 596.77 | <.001 | -1.18 |
| DASS-21 anxiety | 1.42±1.67 | 4.85±2.96 | -20.69 | 687.38 | <.001 | -1.41 |
| DASS-21 stress | 1.16±1.65 | 4.78±2.86 | -22.49 | 694.54 | <.001 | -1.53 |

Note: SPQ-C: Schizotypal Personality Questionnaire- Children; ERQ-CA: Emotion Regulation. Questionnaire for Children and Adolescent; DASS-21: The Depression Anxiety Stress Scales.If Levene's test indicated that the variances were equal across the two groups (p>0.05), we reported the results of the t-test assuming equal variances. Conversely, if Levene's test indicated that the variances were not equal across the two groups (p<0.05), we reported the results of the t-test assuming unequal variances.

**S2.4 Sensitivity Analysis Results for Bayesian network**

**S2.4.1 Figures for Bayesian networks**

**
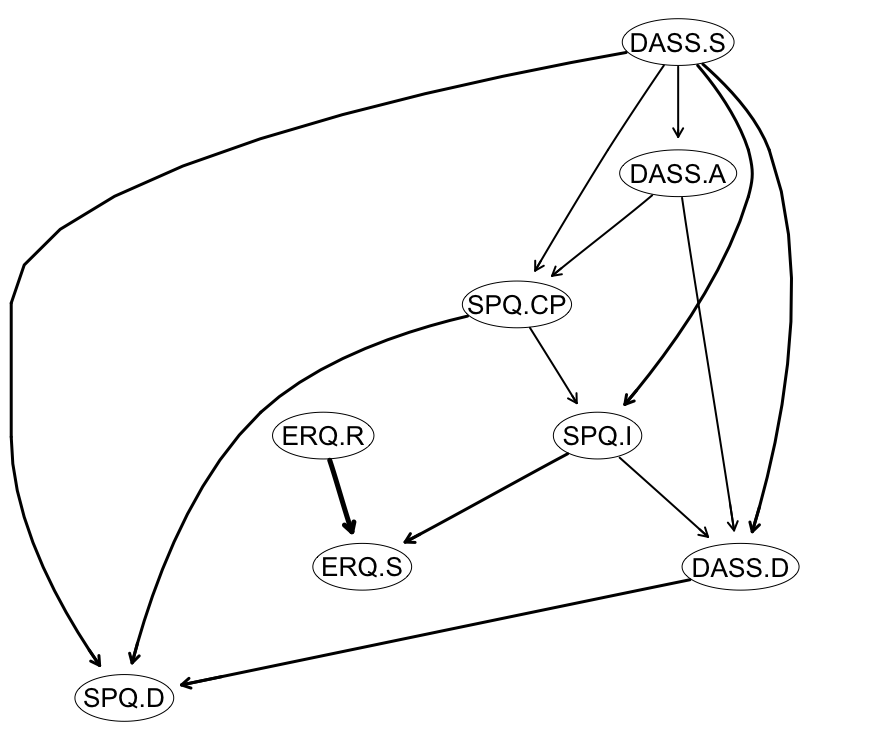
**

**Figure S2.11 A Bayesian network for the whole sample (directed acyclic graph; DAG)**

Note: The edge width corresponds to the direction probabilities, with thicker edges indicating higher direction probabilities. Only edges with probabilities exceeding 85% are displayed in the figure. SPQ.CP = cognitive perceptual dimension of Schizotypal Personality Questionnaire; SPQ.I = interpersonal dimension of Schizotypal Personality Questionnaire; SPQ.D = disorganized dimension of Schizotypal Personality Questionnaire; ERQ.R= reappraisal dimension of Emotion Regulation Questionnaire; ERQ.S= suppression dimension of Emotion Regulation Questionnaire; DASS.D= depression dimension of Depression Anxiety Stress Scales; DASS.A= anxiety dimension of Depression Anxiety Stress Scales; DASS.S= stress dimension of Depression Anxiety Stress Scales;

| (A) Low schizotypy  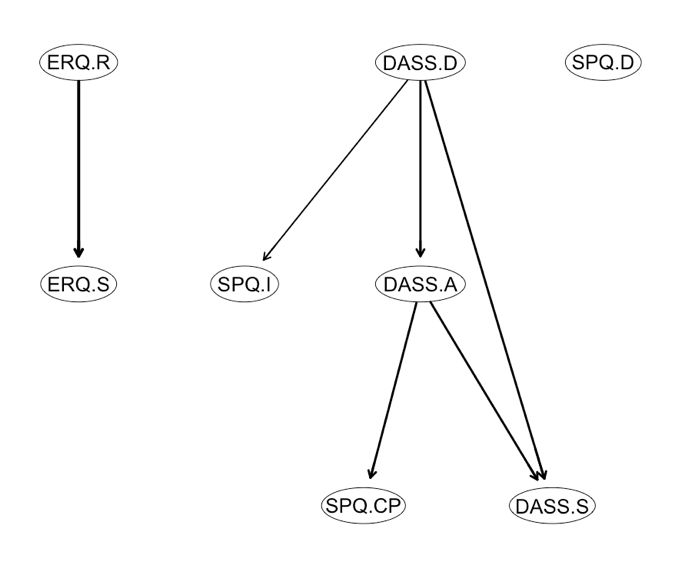 | (B) High schizotypy  **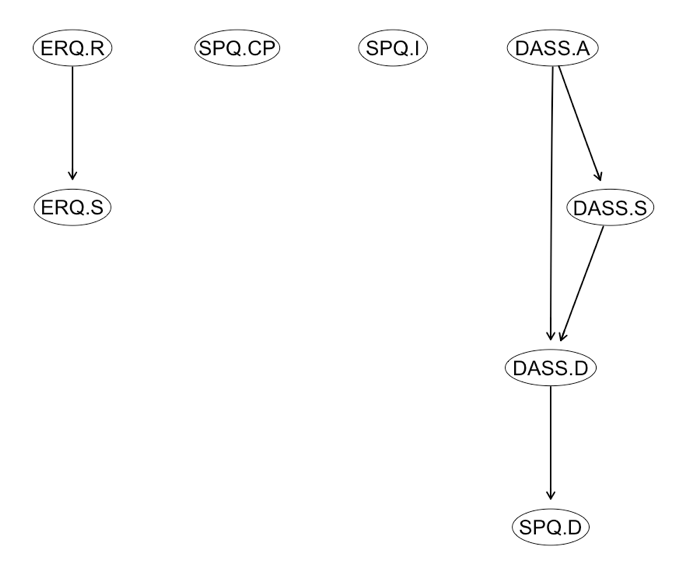** |
| --- | --- |
| **Figure S2.12 (A) Estimated Bayesian networks for the low (left, n=401) and high (right, n=430) schizotypy groups.**  Note: The edge width corresponds to the direction probabilities, with thicker edges indicating higher direction probabilities. Only edges with probabilities exceeding 85% are displayed in the figure. SPQ.CP = cognitive perceptual dimension of Schizotypal Personality Questionnaire; SPQ.I = interpersonal dimension of Schizotypal Personality Questionnaire; SPQ.D = disorganized dimension of Schizotypal Personality Questionnaire; ERQ.R= reappraisal dimension of Emotion Regulation Questionnaire; ERQ.S= suppression dimension of Emotion Regulation Questionnaire; DASS.D= depression dimension of Depression Anxiety Stress Scales; DASS.A= anxiety dimension of Depression Anxiety Stress Scales; DASS.S= stress dimension of Depression Anxiety Stress Scales; | |

| **(A) 9-10 age group**  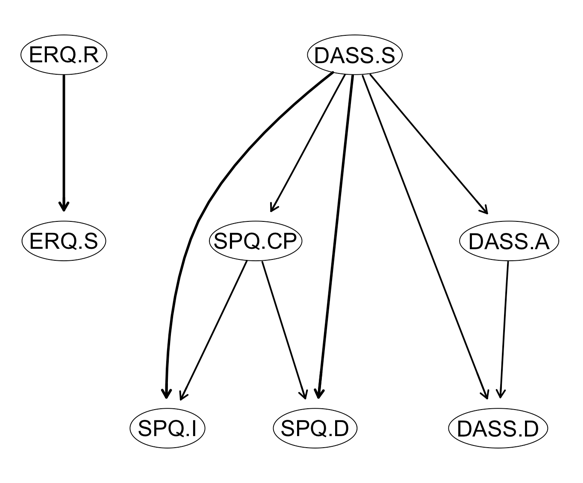 | **(B) 11-12 age group**  **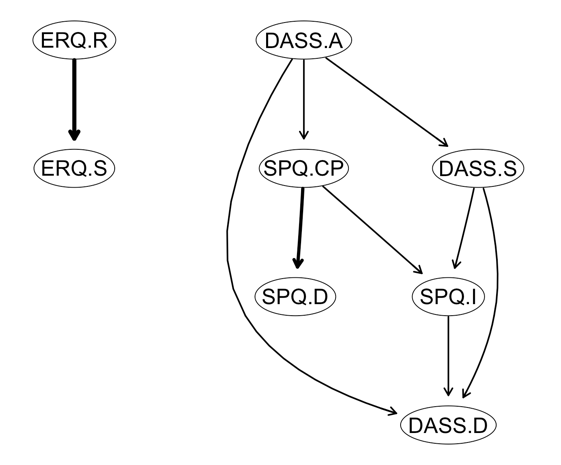** |
| --- | --- |
| **Figure S2.13 (A) Estimated Bayesian networks in children aged 9-10 years (left, n=490) and 11-12 years (right, n=417).**  Note: The edge width corresponds to the direction probabilities, with thicker edges indicating higher direction probabilities. Only edges with probabilities exceeding 85% are displayed in the figure. SPQ.CP = cognitive perceptual dimension of Schizotypal Personality Questionnaire; SPQ.I = interpersonal dimension of Schizotypal Personality Questionnaire; SPQ.D = disorganized dimension of Schizotypal Personality Questionnaire; ERQ.R= reappraisal dimension of Emotion Regulation Questionnaire; ERQ.S= suppression dimension of Emotion Regulation Questionnaire; DASS.D= depression dimension of Depression Anxiety Stress Scales; DASS.A= anxiety dimension of Depression Anxiety Stress Scales; DASS.S= stress dimension of Depression Anxiety Stress Scales; | |

**S2.4.2 Tables for Bayesian networks**

**Table S2.5 Arc strength estimated from the Bayesian networks of the whole sample**

| From | To | Strength | Direction |
| --- | --- | --- | --- |
| ERQ.R | ERQ.S | 1.00 | 0.90 |
| SPQ.CP | SPQ.I | 1.00 | 0.65 |
| SPQ.CP | SPQ.D | 1.00 | 0.75 |
| SPQ.I | ERQ.S | 0.92 | 0.69 |
| SPQ.I | DASS.D | 0.98 | 0.51 |
| DASS.D | ERQ.S | 0.64 | 0.94 |
| DASS.D | SPQ.D | 0.89 | 0.69 |
| DASS.A | SPQ.CP | 1.00 | 0.52 |
| DASS.A | DASS.D | 1.00 | 0.66 |
| DASS.S | ERQ.S | 0.83 | 0.93 |
| DASS.S | SPQ.CP | 0.99 | 0.55 |
| DASS.S | SPQ.I | 1.00 | 0.70 |
| DASS.S | SPQ.D | 0.92 | 0.74 |
| DASS.S | DASS.D | 1.00 | 0.67 |
| DASS.S | DASS.A | 1.00 | 0.52 |

Note: Strength indicates the frequency with which an edge appeared in the bootstrap Bayesian networks. Direction indicates the frequency with which the edge in the Bayesian networks pointed in a given direction. Edges with strength >0.5 and direction >0.5 are shown in the table. In the final DAG, only edges with a strength greater than 85% are included. SPQ.CP = cognitive perceptual dimension of Schizotypal Personality Questionnaire; SPQ.I = interpersonal dimension of Schizotypal Personality Questionnaire; SPQ.D = disorganized dimension of Schizotypal Personality Questionnaire; ERQ.R= reappraisal dimension of Emotion Regulation Questionnaire; ERQ.S= suppression dimension of Emotion Regulation Questionnaire; DASS.D= depression dimension of Depression Anxiety Stress Scales; DASS.A= anxiety dimension of Depression Anxiety Stress Scales; DASS.S= stress dimension of Depression Anxiety Stress Scales;

**Table S2.6 Arc strength estimated from the Bayesian networks of the high schizotypy group.**

| From | To | Strength | Direction |
| --- | --- | --- | --- |
| ERQ.R | ERQ.S | 1.00 | 0.64 |
| SPQ.I | ERQ.S | 0.78 | 0.62 |
| SPQ.I | DASS.D | 0.83 | 0.67 |
| SPQ.I | DASS.S | 0.78 | 0.64 |
| DASS.D | SPQ.D | 0.86 | 0.55 |
| DASS.A | SPQ.CP | 0.64 | 0.68 |
| DASS.A | DASS.D | 1.00 | 0.57 |
| DASS.A | DASS.S | 1.00 | 0.53 |
| DASS.S | ERQ.S | 0.75 | 0.71 |
| DASS.S | SPQ.D | 0.60 | 0.57 |
| DASS.S | DASS.D | 1.00 | 0.55 |

Note: Strength indicates the frequency with which an edge appeared in the bootstrap Bayesian networks. Direction indicates the frequency with which the edge in the Bayesian networks pointed in a given direction. Edges with strength >0.5 and direction >0.5 are shown in the table. In the final DAG, only edges with a strength greater than 85% are included. SPQ.CP = cognitive perceptual dimension of Schizotypal Personality Questionnaire; SPQ.I = interpersonal dimension of Schizotypal Personality Questionnaire; SPQ.D = disorganized dimension of Schizotypal Personality Questionnaire; ERQ.R= reappraisal dimension of Emotion Regulation Questionnaire; ERQ.S= suppression dimension of Emotion Regulation Questionnaire; DASS.D= depression dimension of Depression Anxiety Stress Scales; DASS.A= anxiety dimension of Depression Anxiety Stress Scales; DASS.S= stress dimension of Depression Anxiety Stress Scales;

**Table S2.7 Arc strength estimated from the Bayesian networks of the low schizotypy group.**

| From | To | Strength | Direction |
| --- | --- | --- | --- |
| ERQ.R | ERQ.S | 1.00 | 0.85 |
| DASS.D | ERQ.S | 0.71 | 0.96 |
| DASS.D | SPQ.I | 0.94 | 0.53 |
| DASS.D | DASS.A | 1.00 | 0.57 |
| DASS.D | DASS.S | 1.00 | 0.57 |
| DASS.A | SPQ.CP | 0.92 | 0.56 |
| DASS.A | DASS.S | 1.00 | 0.56 |

Note: Strength indicates the frequency with which an edge appeared in the bootstrap Bayesian networks. Direction indicates the frequency with which the edge in the Bayesian networks pointed in a given direction. Edges with strength >0.5 and direction >0.5 are shown in the table. In the final DAG, only edges with a strength greater than 85% are included. SPQ.CP = cognitive perceptual dimension of Schizotypal Personality Questionnaire; SPQ.I = interpersonal dimension of Schizotypal Personality Questionnaire; SPQ.D = disorganized dimension of Schizotypal Personality Questionnaire; ERQ.R= reappraisal dimension of Emotion Regulation Questionnaire; ERQ.S= suppression dimension of Emotion Regulation Questionnaire; DASS.D= depression dimension of Depression Anxiety Stress Scales; DASS.A= anxiety dimension of Depression Anxiety Stress Scales; DASS.S= stress dimension of Depression Anxiety Stress Scales;

**Table S2.8 Arc strength estimated from the Bayesian networks of the 9-10 age group.**

| From | To | Strength | Direction |
| --- | --- | --- | --- |
| ERQ.R | ERQ.S | 1.00 | 0.75 |
| SPQ.CP | SPQ-I | 1.00 | 0.60 |
| SPQ.CP | SPQ-D | 0.98 | 0.60 |
| SPQ.I | ERQ.S | 0.60 | 0.61 |
| DASS.D | SPQ.I | 0.83 | 0.55 |
| DASS.D | SPQ.D | 0.84 | 0.64 |
| DASS.A | SPQ.CP | 0.68 | 0.60 |
| DASS.A | DASS.D | 0.99 | 0.55 |
| DASS.S | ERQ.S | 0.63 | 0.68 |
| DASS.S | SPQ.CP | 0.96 | 0.62 |
| DASS.S | SPQ.I | 0.97 | 0.69 |
| DASS.S | SPQ.D | 0.86 | 0.68 |
| DASS.S | DASS.D | 1.00 | 0.60 |
| DASS.S | DASS.A | 1.00 | 0.53 |

Note: Strength indicates the frequency with which an edge appeared in the bootstrap Bayesian networks. Direction indicates the frequency with which the edge in the Bayesian networks pointed in a given direction. Edges with strength >0.5 and direction >0.5 are shown in the table. In the final DAG, only edges with a strength greater than 85% are included. SPQ.CP = cognitive perceptual dimension of Schizotypal Personality Questionnaire; SPQ.I = interpersonal dimension of Schizotypal Personality Questionnaire; SPQ.D = disorganized dimension of Schizotypal Personality Questionnaire; ERQ.R= reappraisal dimension of Emotion Regulation Questionnaire; ERQ.S= suppression dimension of Emotion Regulation Questionnaire; DASS.D= depression dimension of Depression Anxiety Stress Scales; DASS.A= anxiety dimension of Depression Anxiety Stress Scales; DASS.S= stress dimension of Depression Anxiety Stress Scales;

**Table S2.9 Arc strength estimated from the Bayesian networks of the 11-12 age group.**

| From | To | Strength | Direction |
| --- | --- | --- | --- |
| ERQ.R | ERQ.S | 1.00 | 0.94 |
| SPQ.C | SPQ.I | 1.00 | 0.62 |
| SPQ.C | SPQ.D | 0.96 | 0.80 |
| SPQ.I | ERQ.S | 0.83 | 0.85 |
| SPQ.I | DASS.D | 0.95 | 0.58 |
| DASS.A | SPQ.C | 1.00 | 0.52 |
| DASS.A | DASS.D | 1.00 | 0.67 |
| DASS.A | DASS.S | 1.00 | 0.60 |
| DASS.S | ERQ.S | 0.71 | 0.92 |
| DASS.S | SPQ.C | 0.60 | 0.51 |
| DASS.S | SPQ.I | 0.93 | 0.53 |
| DASS.S | SPQ.D | 0.68 | 0.80 |
| DASS.S | DASS.D | 1.00 | 0.58 |

Note: Strength indicates the frequency with which an edge appeared in the bootstrap Bayesian networks. Direction indicates the frequency with which the edge in the Bayesian networks pointed in a given direction. Edges with strength >0.5 and direction >0.5 are shown in the table. In the final DAG, only edges with a strength greater than 85% are included. SPQ.CP = cognitive perceptual dimension of Schizotypal Personality Questionnaire; SPQ.I = interpersonal dimension of Schizotypal Personality Questionnaire; SPQ.D = disorganized dimension of Schizotypal Personality Questionnaire; ERQ.R= reappraisal dimension of Emotion Regulation Questionnaire; ERQ.S= suppression dimension of Emotion Regulation Questionnaire; DASS.D= depression dimension of Depression Anxiety Stress Scales; DASS.A= anxiety dimension of Depression Anxiety Stress Scales; DASS.S= stress dimension of Depression Anxiety Stress Scales;

Reference:

Jones P. R. (2019). A note on detecting statistical outliers in psychophysical data. *Attention, perception & psychophysics*, *81*(5), 1189–1196. https://doi.org/10.3758/s13414-019-01726-3
