# Supplementary material for: Relationship Between Schizotypal Traits, Emotion Regulation, and Negative Affect in Children: A Network Analysis
Source: Schizophr Bull. 2025 Mar 4;51(Suppl 2):S226–37. doi: 10.1093/schbul/sbae172 (PMC11879503; doi:10.1093/schbul/sbae172)
Supplement: sbae172_suppl_Supplementary_Materials [file sbae172_suppl_supplementary_materials.zip › Supplementary materials_3 07_08_24.docx]

## ----------------------------------- Supplementary R Scripts ----------------------------------- ##

## Article: Relationship between schizotypal traits, emotion regulation and negative affect in children: A network analysis

## -----------------------------load data and prepare for analysis------------------------------##

library(readxl)

data<-read_excel("/Users/rqfile/Desktop/data_06_17/dataprocess_final/data_used_for_analysis/new_processed_data.xlsx")

## Participants above the age of 13 was exluded, as 13 years is recognized as adolescence according to WHO recommendations.

data <- data[data$age < 13, ]

## Due to the fact that our participants are from grades four to six, data from individuals below the age of 7 is a result of input errors.

data <- data[data$age > 8, ]

summary(data)

## Extracting nodes for network analysis for the whole-sample network and gender networks

mydata<-data[, c("ERQ.R","ERQ.S","SPQ.C","SPQ.I","SPQ.D","DASS.D", "DASS.A", "DASS.S")]

databoy_filtered <- data[data$cgender == 1, ]

datagirl_filtered <- data[data$cgender == 2, ]

databoy<-databoy_filtered[, c("ERQ.R","ERQ.S","SPQ.C","SPQ.I","SPQ.D","DASS.D", "DASS.A", "DASS.S")]

datagirl<-datagirl_filtered[, c("ERQ.R","ERQ.S","SPQ.C","SPQ.I","SPQ.D","DASS.D", "DASS.A", "DASS.S")]

names(mydata)[names(mydata) == "SPQ.C"] <- "SPQ.CP"

names(databoy)[names(databoy) == "SPQ.C"] <- "SPQ.CP"

names(datagirl)[names(datagirl) == "SPQ.C"] <- "SPQ.CP"

## -----------Import libraries (Please make sure you have installed these packages first) --------##

library(bootnet) # network stability and accuracy

library(semPlot)

library(lavaanPlot)

library(mgm)# predictability

library(igraph)

library(qgraph)# estimate network

library(networktools)# EI, expected influence

library(dplyr)

library(ggplot2)

library(Hmisc)

library(bootnet)

library(qgraph)

library(networktools)

library(NetworkComparisonTest)

library(mgm)

library(igraph)

library(DiagrammeRsvg)

library(moments)

library(gridExtra)

## ---------------------------------------------------Distribution test ------------------------------------------------##

##Performing a distribution test so we can choose an appropriate correlation analysis method.

calculate_skew_kurt <- function(variable) {

skew <- skewness(variable)

kurt <- kurtosis(variable)

return(c(skewness = skew, kurtosis = kurt))}

##apply the calculate_skew_kurt function to every column of the data and stored results

skresult_data <- apply(mydata, 2, calculate_skew_kurt)

skresult_databoy <- apply(databoy, 2, calculate_skew_kurt)

skresult_datagirl <- apply(datagirl, 2, calculate_skew_kurt)

### Labels of network

mynames <- c("Reappraisal","Suppression","Cognitive perceptual","Interpersonal","Disorganised","Depression", "Anxiety", "Stress")

L<-c("ERQ.R","ERQ.S","SPQ.CP","SPQ.I","SPQ.D","DASS.D", "DASS.A", "DASS.S")

clusters<-list("ERQ"=c(1:2),"SPQ"=c(3:5),"DASS"=c(6:8))

###translate to z-score.Z-score transformation allows for the comparison of variables on a common scale by eliminating differences in their units of measurement,which facilitates better comparison and interpretation of the relationships between different variables.

scaled_data <- scale(mydata)

scaled_boydata <- scale(databoy)

scaled_girldata <- scale(datagirl)

##distribution test again

##apply the calculate_skew_kurt function to every column of the data and stored results

skresult_scaledata <- apply(scaled_data, 2, calculate_skew_kurt)

skresult_scaledataboy <- apply(scaled_boydata, 2, calculate_skew_kurt)

skresult_scaledatagirl <- apply(scaled_girldata, 2, calculate_skew_kurt)

## ----------------------------------------------------Part 1: Network estimates, visualization, centrality estimates: Regularised partial correlation networks-----------------------------------------------------#

##network estimation, due to the non-normality of the data, the Spearman's method is being used.##

mynetwork<-estimateNetwork(scaled_data,default="EBICglasso",corMethod="spearman", tuning=0.5)

boynetwork<-estimateNetwork(scaled_boydata,default="EBICglasso",corMethod="spearman", tuning=0.5)

girlnetwork<-estimateNetwork(scaled_girldata,default="EBICglasso",corMethod="spearman", tuning=0.5)

##save correlation

Weights <- getWmat(mynetwork)

Weights_boy <- getWmat(boynetwork)

Weights_girl <- getWmat(girlnetwork)

###Predictability of each node calculated ###

##the whole sample

mydata2 <- as.matrix(scaled_data)

p <- ncol(mydata2)

set.seed(1)

fit_obj_data <- mgm(data=mydata2, type = rep('g', p), level = rep(1, p), ruleReg = 'AND',

k=2)

pred_obj_data<- predict(object = fit_obj_data, data = mydata2, errorCon = 'R2')

pred_obj_data$error

meanpredictibility<-mean(pred_obj_data$error$R2)

##the boy subgroup

databoy2 <- as.matrix(scaled_boydata)

p <- ncol(databoy2)

set.seed(1)

fit_obj_boydata <- mgm(data=databoy2, type = rep('g', p), level = rep(1, p), ruleReg = 'AND',

k=2)

pred_obj_boydata <- predict(object = fit_obj_boydata, data = databoy2, errorCon = 'R2')

#the girl subgroup

datagirl2 <- as.matrix(scaled_girldata)

p <- ncol(datagirl2)

set.seed(1)

fit_obj_girldata <- mgm(data=datagirl2, type = rep('g', p), level = rep(1, p), ruleReg = 'AND',

k=2)

pred_obj_girldata <- predict(object = fit_obj_girldata, data = datagirl2, errorCon = 'R2')

###------------------------ Network visualization --------------------###

gr1<-list(c(1,2), c(3,4,5),c(6,7,8))

names(gr1)<-c( "ERQ Nodes", "SPQ Nodes","DASS Nodes")

##whole-sample network

myplot<-plot(mynetwork, labels=L, nodeNames=mynames, pie=pred_obj_data$errors$R2, legend.cex=.6, pieColor="gray40",groups=gr1, palette="pastel",layout="spring", label.scale.equal = TRUE,label.scale = TRUE,edge.labels = TRUE)

#plot boynetwork

myplotboy<-plot(boynetwork, labels=L, nodeNames=mynames, pie=pred_obj_boydata$errors$R2, legend.cex=.6, pieColor="gray40",groups=gr1, palette="pastel",layout="circle", label.scale.equal = TRUE,label.scale = TRUE,edge.labels = TRUE)

#plot girlnetwork

myplotgirl<-plot(girlnetwork, labels=L, nodeNames=mynames, pie=pred_obj_girldata$errors$R2, legend.cex=.6, pieColor="gray40",groups=gr1,palette="pastel",layout="circle", label.scale.equal = TRUE,label.scale = TRUE,edge.labels = TRUE)

###Centrality estiamtes###

centralityPlot_whole<-centralityPlot(mynetwork, include ="all",scale=c("z-scores"),orderBy="Strength")

centralityTable_whole<-centralityTable(mynetwork)

centralityPlot_girl<-centralityPlot(list(girlnetwork), include ="all",orderBy="Strength")

centralityPlot_boy<-centralityPlot(list(boynetwork), include ="all",orderBy="Strength")

centralityTable_girl<-centralityTable(girlnetwork)

centralityTable_boy<-centralityTable(boynetwork)

###--------------------------Network stability----------------------###

###edge accuracy####

boot1<-bootnet(mynetwork,nBoots=2500,nCores = 8,statistics

=c("strength","closeness","betweenness","edge","expectedInfluence"))

plot(boot1,labels = TRUE,order = "sample")

bootgirl<-bootnet(girlnetwork,nBoots=2500,nCores = 8)

plot(bootgirl,labels = TRUE, order = "sample")

bootboy<-bootnet(boynetwork,nBoots=2500,nCores = 8)

plot(bootboy,labels = TRUE, order = "sample")

### CS coefficient, centraility stability###

bootCASE<-bootnet(mynetwork, default="EBICglasso", nCores = 8, nBoots = 2500,

statistics = c("strength","expectedInfluence", "closeness", "betweenness"),type = "case")

CentralStability<-corStability(bootCASE)

bootCASEgirl<-bootnet(girlnetwork, default="EBICglasso", nCores = 8, nBoots = 2500,

statistics = c("strength","expectedInfluence", "closeness", "betweenness"),type = "case")

bootCASEboy<-bootnet(boynetwork, default="EBICglasso", nCores = 8, nBoots = 2500,

statistics = c("strength","expectedInfluence", "closeness", "betweenness"),type = "case")

### Compute and plot CS-coefficients ###

CentralStability<-corStability(bootCASE)

plot(bootCASE,statistics ="all")

CentralStabilitygirl<-corStability(bootCASEgirl)

plot(bootCASEgirl,statistics ="all")

CentralStability<-corStability(bootCASEboy)

plot(bootCASEboy,statistics ="all")

### Plot significant differences of edge weights ###

plot(boot1, "edge", plot = "difference", onlyNonZero = TRUE, cex.axis = 0.5,

order = "sample")

### Plot significant differences of node strength###

plot1 <- plot(boot1, "strength", main="Strength")

plot2 <- plot(boot1, "expectedInfluence", main="Expected Influence")

plot3 <- plot(boot1, "closeness", main="Closeness")

plot4 <- plot(boot1, "betweenness", main="Betweenness")

grid.arrange(plot1, plot2, plot3,plot4, ncol=2)

## ----------------------------------------------Part 2: Bridge centrality --------------------------------------------#

community_structure <- c(rep("ERQ", 2), rep("SPQ",3),rep('DASS',3))

bridge_centrality<-bridge(myplot,communities = community_structure, directed = NULL,nodes = NULL,normalize = FALSE)

plot(bridge_centrality, include=c("Bridge Strength", "Bridge Betweenness","Bridge Expected Influence (1-step)","Bridge Closeness"), zscore=TRUE)

## Select the top 80th percentile bridge centrality:

bridge_strength <- bridge_centrality$`Bridge Strength`

bridge_Expected_influence <- bridge_centrality$`Bridge Expected Influence (1-step)`

bridge_betweenness <- bridge_centrality$`Bridge Betweenness`

bridge_closeness <- bridge_centrality$`Bridge Closeness`

top_bridges <- names(bridge_strength[bridge_strength>quantile(bridge_strength, probs=0.80, na.rm=TRUE)])

top_bridges2 <- names(bridge_Expected_influence[bridge_Expected_influence>quantile(bridge_Expected_influence, probs=0.80, na.rm=TRUE)])

top_bridges3 <- names(bridge_betweenness[bridge_betweenness>quantile(bridge_betweenness, probs=0.80, na.rm=TRUE)])

top_bridges4 <- names(bridge_closeness[bridge_closeness>quantile(bridge_closeness, probs=0.80, na.rm=TRUE)])

### Plot stability of bridge centralities###

bootCASE2<-bootnet(mynetwork, default="EBICglasso", nCores = 8, nBoots = 2500,

statistics ="all" ,type="case",communities = community_structure)

plot(bootCASE2, statistics= c("bridgeStrength","bridgeCloseness","bridgeBetweenness","bridgeExpectedInfluence"))

bootbridge<-bootnet(mynetwork,nBoots=2500,nCores = 8,statistics= c("bridgeStrength","bridgeCloseness","bridgeBetweenness","bridgeExpectedInfluence"))

plot5 <- plot(bootbridge, "bridgeStrength", main = "bridgeStrength",plot = "difference")

plot6 <- plot(bootbridge, "bridgeExpectedInfluence", main = "bridgeExpectedInfluence",plot = "difference")

plot7 <- plot(bootbridge, "bridgeCloseness", main = "bridgeCloseness",plot = "difference")

plot8 <- plot(bootbridge, "bridgeBetweenness", main = "bridgeBetweenness",plot = "difference")

grid.arrange(plot5, plot6, plot7, plot8, ncol = 2)

### Plot significant differences of node strength###

## -------------------------------------------Part 3: Bayesian network of whole sample--------------------------------------------#

##-- Net1: build the average network using a 0.85 threshold (Sachs et al., 2005, Science)----#

###Load packages ###

require("bnlearn") ## Bayesian network package

require("corrplot") ## correlation matrix plots

library("Rgraphviz") ## used for visualizing Bayesian networks

###------------Net1: Whole sample DAG--------------###

netdata <- as.data.frame(apply(scaled_data, 2, as.numeric)) ## convert to numerics

Bayeisannetwork <- hc(netdata, restart = 50, perturb = 100) ## hc gives directed graph

set.seed(123)

bootnet <- boot.strength(netdata, R = 1000, algorithm = "hc", algorithm.args = list(restart = 5, perturb = 10), debug = TRUE)

## filter the ones with a strength larger than 0.85 and a direction probability > 0.5

bootwhole<-bootnet[bootnet$strength > 0.85 & bootnet$direction > 0.5, ]

avgnetwhole <- averaged.network(bootnet, threshold = 0.85)

## edge strenghts are determined by direction probability

astrwhole <- bootwhole ## table with direction probabilities

astrwhole $strength <- astrwhole$direction ## use the direction probabilities for edge width

dev.new()

strength.plot(avgnetwhole, astrwhole, shape = "ellipse",threshold = 0.50)## thick arrows indicate high directional probabilties, thin arrows low directional probabilities

## -------------------------------------------Part 4: Network comparisons --------------------------------------------##

###-------------------------- Gender effect----------------------###

compnetwork <- NCT(girlnetwork,boynetwork, it=10000,

test.centrality=TRUE,nodes="all",centrality = c("strength", "expectedInfluence","closeness"),

progressbar=TRUE, test.edges = TRUE,

edges='all',p.adjust.methods = c("fdr"))

summary(compnetwork)

plot(compnetwork, what="network")

plot(compnetwork, what="strength")

#Compare centrality for each node

compnetwork$diffcen.pval

#Compare global strength

compnetwork$glstrinv.pval

#Compare structure

compnetwork$nwinv.pval

compnetwork$einv.pvals

###-------------------------- Age effect----------------------###

library(dplyr)

library(bootnet)

agedata<-data[, c("age","ERQ.R","ERQ.S","SPQ.C","SPQ.I","SPQ.D","DASS.D", "DASS.A", "DASS.S")]

data_9 <- agedata %>% filter(agedata$age == 9 | agedata$age == 10)

data_11 <- agedata %>% filter(agedata$age == 11 | agedata$age == 12)

scale_9 <- data_9[, c("ERQ.R","ERQ.S","SPQ.C","SPQ.I","SPQ.D","DASS.D", "DASS.A", "DASS.S")]

scale_11 <- data_11[, c("ERQ.R","ERQ.S","SPQ.C","SPQ.I","SPQ.D","DASS.D", "DASS.A", "DASS.S")]

scale_9 <- scale(scale_9)

scale_11 <- scale(scale_11)

names(scale_9)[names(scale_9) == "SPQ.C"] <- "SPQ.CP"

names(scale_11)[names(scale_11) == "SPQ.C"] <- "SPQ.CP"

mynetwork9 <- estimateNetwork(scale_9, default = "EBICglasso", corMethod = "spearman", tuning = 0.5)

mynetwork11 <- estimateNetwork(scale_11, default = "EBICglasso", corMethod = "spearman", tuning = 0.5)

##--------------Age network comparison--------------##

comparison_9_11 <- NCT(mynetwork9, mynetwork11, it = 10000,test.centrality=TRUE,nodes="all",centrality = c("strength", "expectedInfluence","closeness"),

progressbar=TRUE, test.edges = TRUE,

edges='all',p.adjust.methods = c("fdr"))

myplot9<-plot(mynetwork9, labels=L, nodeNames=mynames, plegend.cex=.6, pieColor="gray40",groups=gr1, palette="pastel",layout="circle", label.scale.equal = TRUE,label.scale = TRUE,edge.labels = TRUE)

myplot11<-plot(mynetwork11, labels=L, nodeNames=mynames, plegend.cex=.6, pieColor="gray40",groups=gr1, palette="pastel",layout="circle", label.scale.equal = TRUE,label.scale = TRUE,edge.labels = TRUE)

#Compare centrality for each node

comparison_9_11$diffcen.pval

#Compare global strength

comparison_9_11$glstrinv.pval

#Compare structure

comparison_9_11$nwinv.pval

comparison_9_11$einv.pvals

comparison_9_11$einv.pvals$`p-value` <- as.numeric(as.character(comparison_9_11$einv.pvals$`p-value`))

# Filter rows where 'p-value' is less than 0.05

significant_pvals <- comparison_9_11$einv.pvals[comparison_9_11$einv.pvals$`p-value` < 0.05, ]

##-------------------Age DAG------------------------##

#### 9-10 age group DAG####

netdata9 <- as.data.frame(apply(scale_9, 2, as.numeric)) ## convert to numerics

Bayeisannetwork9 <- hc(netdata9, restart = 50, perturb = 100) ## hc gives directed graph

Bayeisannetwork9

bnlearn::score(Bayeisannetwork9, data = netdata9) ## global network score

set.seed(123)

bootnet9 <- boot.strength(netdata9, R = 1000, algorithm = "hc", algorithm.args = list(restart = 5, perturb = 10), debug = TRUE)

## filter the ones with a strength larger than 0.85 and a direction probability > 0.5

bootnet19<-bootnet9[bootnet9$strength > 0.85 & bootnet9$direction > 0.5, ]

avgnet19 <- averaged.network(bootnet9, threshold = 0.85)

avgnet19

## edge strenghts are determined by direction probability

astr9 <- bootnet19 ## table with direction probabilities

astr9$strength <- astr9$direction ## use the direction probabilities for edge width

strength.plot(avgnet19, astr9, shape = "ellipse",threshold = 0.50)## thick arrows indicate high directional probabilties, thin arrows low directional probabilities

#### 11-12 age group DAG####

netdata11 <- as.data.frame(apply(scale_11, 2, as.numeric))

Bayeisannetwork11 <- hc(netdata11, restart = 50, perturb = 100)

Bayeisannetwork11

bnlearn::score(Bayeisannetwork11, data = netdata11)

set.seed(123)

bootnet11 <- boot.strength(netdata11, R = 1000, algorithm = "hc", algorithm.args = list(restart = 5, perturb = 10), debug = TRUE)

bootnet11 <- bootnet11[bootnet11$strength > 0.85 & bootnet11$direction > 0.5, ]

# Create an averaged network with a threshold of 0.5

avgnet11 <- averaged.network(bootnet11, threshold = 0.85)

avgnet11

# Use the direction probabilities for edge width in the strength plot

astr_11 <- bootnet11

astr_11$strength <- astr_11$direction # Use direction probabilities for edge width

# Plot the edge strengths using the averaged network and direction probabilities

strength.plot(avgnet11, astr_11, shape = "ellipse", threshold = 0.50)

###------------------------Schizotypy high/low effect-------------------------###

##Split data into high and low schizotypy subgroups###

SPQdata<-data[, c("SPQ.C","SPQ.I","SPQ.D")]

SPQ_total<-rowSums(SPQdata)

mydata2 <- cbind(mydata, SPQ_total)

thre_l <- quantile(mydata2$SPQ_total, 0.5)

thre_h <- quantile(mydata2$SPQ_total, 0.5)

top_h <- mydata2[mydata2$SPQ_total > thre_h, ]

bot_l <- mydata2[mydata2$SPQ_total < thre_l, ]

top_h<-top_h[, c("ERQ.R","ERQ.S","SPQ.CP","SPQ.I","SPQ.D","DASS.D", "DASS.A", "DASS.S")]

bot_l<-bot_l[, c("ERQ.R","ERQ.S","SPQ.CP","SPQ.I","SPQ.D","DASS.D", "DASS.A", "DASS.S")]

scaled_h <- scale(top_h)

scaled_l <- scale(bot_l)

##---------------Schizotypy high/low network comparison--------------##

mynetwork_high <- estimateNetwork(scaled_h, default = "EBICglasso", corMethod = "spearman", tuning = 0.5)

mynetwork_low <- estimateNetwork(scaled_l, default = "EBICglasso", corMethod = "spearman", tuning = 0.5)

comparisonhigh_low <- NCT(mynetwork_high, mynetwork_low, it = 10000,test.centrality=TRUE,nodes="all",centrality = c("strength", "expectedInfluence","closeness"),

progressbar=TRUE, test.edges = TRUE,

edges='all',p.adjust.methods = c("fdr"))

myplot_high<-plot(mynetwork_high, labels=L, nodeNames=mynames, plegend.cex=.6, pieColor="gray40",groups=gr1, palette="pastel",layout="circle", label.scale.equal = TRUE,label.scale = TRUE,edge.labels = TRUE)

myplot_low<-plot(mynetwork_low, labels=L, nodeNames=mynames, plegend.cex=.6, pieColor="gray40",groups=gr1, palette="pastel",layout="circle", label.scale.equal = TRUE,label.scale = TRUE,edge.labels = TRUE)

#Compare centrality for each node

#Compare centrality for each node

comparisonhigh_low$diffcen.pval

#Compare global strength

comparisonhigh_low$glstrinv.pval

#Compare structure

comparisonhigh_low$nwinv.pval

comparisonhigh_low$einv.pvals

# Convert 'p-value' to numeric

comparisonhigh_low$einv.pvals$`p-value` <- as.numeric(as.character(comparisonhigh_low$einv.pvals$`p-value`))

# Filter rows where 'p-value' is less than 0.05

significant_pvals <- comparisonhigh_low$einv.pvals[comparisonhigh_low$einv.pvals$`p-value` < 0.05, ]

###----------------Schizotypy high/low DAGs--------------###

##high schizotypy subgroups DAG##

netdatah <- as.data.frame(apply(scaled_h, 2, as.numeric)) ## convert to numerics

Bayeisannetworkh <- hc(netdatah, restart = 50, perturb = 100) ## hc gives directed graph

Bayeisannetworkh

bnlearn::score(Bayeisannetworkh, data = netdatah) ## global network score

set.seed(123)

bootneth <- boot.strength(netdatah, R = 1000, algorithm = "hc", algorithm.args = list(restart = 5, perturb = 10), debug = TRUE)

## filter the ones with a strength larger than 0.85 and a direction probability > 0.5

bootnet1h<-bootneth[bootneth$strength > 0.85 & bootneth$direction > 0.5, ]

avgnet1h <- averaged.network(bootneth, threshold = 0.85)

avgnet1h

## edge strenghts are determined by direction probability

astrh <- bootnet1h ## table with direction probabilities

astrh$strength <- astrh$direction ## use the direction probabilities for edge width

strength.plot(avgnet1h, astrh, shape = "ellipse",threshold = 0.50)## thick arrows indicate high directional probabilties, thin arrows low directional probabilities

##low schizotypy subgroups DAG##

netdatal <- as.data.frame(apply(scaled_l, 2, as.numeric)) ## convert to numerics

Bayeisannetworkl <- hc(netdatal, restart = 50, perturb = 100) ## hc gives directed graph

Bayeisannetworkl

bnlearn::score(Bayeisannetworkl, data = netdatal) ## global network score

set.seed(123)

bootnetl <- boot.strength(netdatal, R = 1000, algorithm = "hc", algorithm.args = list(restart = 5, perturb = 10), debug = TRUE)

## filter the ones with a strength larger than 0.85 and a direction probability > 0.5

bootnet1l<-bootnetl[bootnetl$strength > 0.85 & bootnetl$direction > 0.5, ]

avgnet1l <- averaged.network(bootnet1l, threshold = 0.85)

avgnet1l

## edge strenghts are determined by direction probability

astrl <- bootnet1l ## table with direction probabilities

astrl$strength <- astrl$direction ## use the direction probabilities for edge width

strength.plot(avgnet1l, astrl, shape = "ellipse",threshold = 0.30)## thick arrows indicate high directional probabilties, thin arrows low directional probabilities
